# Antibiotic consumption patterns in acute care hospitals: an integrated analysis using regression modelling combining data from two surveillance systems, Germany, 2022

**DOI:** 10.2807/1560-7917.ES.2025.30.45.2500560

**Published:** 2025-11-13

**Authors:** Karin Gröschner, Winfried V Kern, Tim Eckmanns, Birgitta Schweickert, Gesche Först, Ulrike Georgi, Marcel Feig, Michaela Steib-Bauert, Niklas Willrich, Katja de With

**Affiliations:** 1 Department of Infectious Disease Epidemiology, Robert Koch Institute, Berlin, Germany; 2 Division of Infectious Diseases, Department of Medicine II, University Hospital and Medical Centre, and Faculty of Medicine, Albert-Ludwigs-University, Freiburg, Germany; 3 Clinical Pharmacy, Institute of Pharmaceutical Sciences, Faculty of Chemistry and Pharmacy, Albert-Ludwigs-University, Freiburg, Germany; 4 ADKA-Bundesverband Deutscher Krankenhausapotheker e.V., Berlin, Germany; 5 Pharmacy Service of Clinical Centre Chemnitz, Chemnitz, Germany; 6 Department for Method Development, Research Infrastructure and Information Technology, Robert Koch Institute, Berlin, Germany; 7 Institute of Infectious Diseases, University Hospital Carl Gustav Carus, Technical University, Dresden, Germany; 8 DGI-Deutsche Gesellschaft für Infektiologie e.V., Berlin, Germany

**Keywords:** antibiotic consumption, Germany, acute care hospitals, regression modelling, surveillance, regional differences

## Abstract

**BACKGROUND:**

To enhance antibiotic stewardship and effectively address antimicrobial resistance (AMR), better understanding of subnational antibiotic consumption patterns is essential.

**AIM:**

We aimed to assess antibiotic consumption in Germany using data from 2022 and integrated from two surveillance systems, focusing on regional differences by examining non-university acute care hospitals.

**METHODS:**

We used pharmacy dispensing data from 525 regional or local hospitals and 35 university hospitals, covering 46.5 million patient days (PD), nearly half of all occupied bed days nationwide, to calculate antibiotic use densities (AUD) for systemic antibiotics, expressed as World Health Organization (WHO) ATC/DDD (Anatomical Therapeutic Chemical/Defined Daily Dose) per 100 patient days (DDD/100 PD). The analysis primarily focused on consumption patterns in non-university hospitals, assessing key antibiotic groups through mixed-effects regression. For sensitivity analyses, we employed hospital-adapted daily dose definitions.

**RESULTS:**

Pooled AUD for participating non-university hospitals was 51.8 DDD/100 PD, with aminopenicillins/beta-lactamase inhibitors being the most prescribed group. Regression analyses, adjusted for hospital size and ward type/admitting specialty, indicated notable regional variation. We identified statistically significant differences in antibiotic consumption, particularly for beta-lactam antibiotics, fluoroquinolones and tetracyclines. For example, several regions exhibited up to 1.4-fold higher use of first- and second-generation cephalosporins compared with the western reference region.

**CONCLUSION:**

This study highlights substantial regional variation in antibiotic use in German acute care hospitals, underlining the importance of further investigation into influencing factors such as regional guidelines and resistance rates. The methodological approach applied here may serve as a model for other countries interested in analysing regional differences in antibiotic consumption.

**Key public health message**

**What did you want to address in this study and why?**

We aimed to investigate regional variation in hospital antibiotic use across Germany with data from 2022. Such variation is highly relevant for the development and spread of antibiotic resistance. By moving beyond hospital-level analyses and outpatient care studies, our work strengthens the national evidence base and underscores the need for coordinated strategies to ensure prudent antibiotic use across regions.

**What have we learnt from this study?**

Using a large and representative dataset, we provided more robust evidence than previous studies and generated new insights into prescribing patterns, including confirmation that both high-density and high-volume areas contribute to AMR development. Regional variations were notable, with some antibiotic groups showing up to 1.4 times higher use in certain areas, underscoring the need for targeted stewardship.

**What are the implications of your findings for public health?**

Recognising that differences in prescribing practices may reflect local guidelines, resistance rates or healthcare structures, these findings highlight the importance of incorporating regional perspectives into national antimicrobial stewardship strategies. The methodology applied here provides a model for other countries seeking to better understand and address local variation in antibiotic use as part of broader surveillance and stewardship efforts.

## Introduction

Antimicrobial resistance (AMR) has become a global health threat with increasing relevance for mortality, morbidity and medical expenses [1]. Therefore, the targeted and prudent use of antibiotics is a global priority. The World Health Organization (WHO)’s AWaRe (Access, Watch, Reserve) system supports appropriate prescribing [2], with the European Union/European Economic Area (EU/EEA) seeking to increase the share of Access antibiotics to 65% by 2030 [3]. Germany's AMR action plan DART 2030 (Deutsche Antibiotika-Resistenzstrategie) aligns with these efforts, aiming to enhance AMR surveillance and fostering antibiotic stewardship across health sectors [4].

Although most antibiotics are prescribed in the community, including long-term care facilities [5], monitoring hospital use is specifically important given higher antibiotic use densities (AUDs) and the vulnerability of patients in this setting. European data show wide variation in antibiotic use and hospital vs community share, although national data often hide regional differences especially in large countries where hospital data often lack full coverage, may be biased towards public institutions or have uneven geographic distribution [5-8].

Germany’s hospital system comprises around 1,900 facilities and 477,000 beds, with regional bed densities ranging from < 50 to > 70 beds per 10,000 inhabitants [9]. As in other large EU/EEA countries, comprehensive national-level antibiotic consumption data are lacking.

Detecting regional differences in antibiotic prescribing is essential for guiding stewardship interventions but existing approaches, which often require complete datasets, have limited applicability. In this study, we integrated data from the two main German antibiotic consumption surveillance systems — the Antibiotic Consumption Surveillance System (AVS) and the ADKA-if-DGI Surveillance System (German Association of Hospital Pharmacists -Infectiology Freiburg - German Society for Infectious Diseases) [10,11] — and applied regression modelling to assess regional variation in inpatient antibiotic prescribing in 2022. 

## Methods

### Data sources and inclusion criteria

We conducted analyses using data provided by two national antibiotic surveillance systems: AVS [10], coordinated by the Robert Koch Institute since 2014, and the ADKA-if-DGI Surveillance System [11]. Both rely on voluntarily participation by hospitals and collect ward-level data on drug dispensing, admissions and patient days. Participating hospitals receive individual feedback and benchmarking reports on AUD. Detailed descriptions of both systems are available elsewhere [10-12].

**Figure 1:**
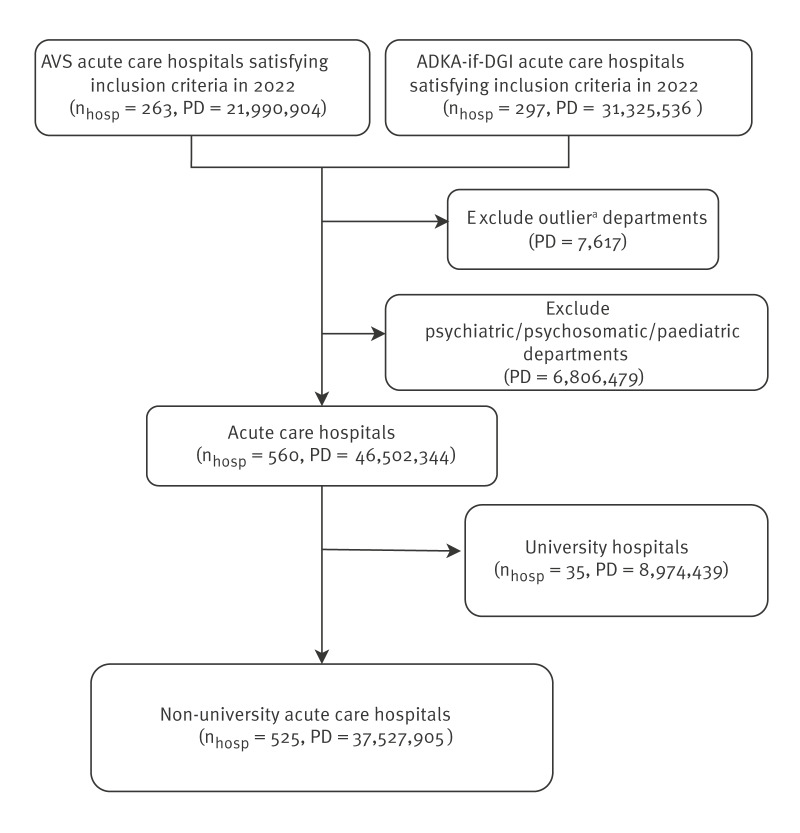
Flow diagram of hospital selection according to inclusion and exclusion criteria, Germany, 2022 (n_hosp_ = 525)

ADKA-if-DGI: German Association of Hospital Pharmacists -Infectiology - German Society for Infectious Diseases- Surveillance System; AVS: Antibiotic Consumption Surveillance System at Robert Koch Institute; DDD: Defined Daily Doses; n_hosp_: number of hospitals; PD: patient days.

^a^ In our dataset, the majority of excluded patient days (~87%) originated from a single gynaecological ward.

In line with our inclusion criteria as shown in Figure 1, we focussed on general acute care hospitals with at least one general medicine department or with two departments, one of which must be surgery, anaesthesiology or internal medicine. We excluded paediatric departments, because of weight-based dosing and distinct prescribing patterns, and psychiatric and psychosomatic departments because high bed-day numbers could underestimate antibiotic consumption relative to hospitals without such departments. We excluded monospecialty hospitals (e.g. geriatrics, neurology), rehabilitation centres and wards with antibiotic use exceeding 500 Defined Daily Doses per 100 patient days (DDD/100 PD), considered outliers. We included university-affiliated hospitals in nationwide descriptive analyses only (see Supplementary Figure S2 for descriptive data on university hospitals), but excluded them from regional analyses due to differing regional assignment criteria to avoid identifiability. We distinguished intensive care units (ICUs) and general wards, classifying ICUs as surgical/interdisciplinary or non-surgical, and general wards by admitting specialty: ‘medical’ (general internal medicine, haematology-oncology, and other non-surgical specialties such as neurology) or ‘surgical’ (general surgery, defined surgical subspecialties, and other non-medical/interdisciplinary wards). See Supplementary Table S1 for the definition of ward groups. 

Inclusion/exclusion criteria* –* Included: general acute care hospitals with at least one general medicine or two departments, one of which must be surgery/anaesthesiology/internal medicine department. Excluded: paediatric, psychiatric, psychosomatic departments, monospecialty hospitals, rehabilitation centres, and outliers (>500 DDD/100 PD). University hospitals were included only in nationwide descriptive analyses. ICUs were classified as surgical/non-surgical; general wards as ‘medical’ or ‘surgical’.

Additional hospital-level information included bed count (< 200, 200–399, 400–799, ≥ 800), university affiliation (yes/no), and region (North-west (NW), North-east (NE], West (W), Centre/East (CE), South-west (SW) and South-east (SE)) for non-university hospitals. The definition of regions corresponded to one or more federal states, accounting for similar population sizes. See Supplementary Figure S1 for the definition of regions.

The dataset comprised 560 hospitals, including 263 hospitals participating in the AVS Surveillance System and 297 hospitals participating in the ADKA-if-DGI Surveillance System, each of which had submitted ward-level antibiotic consumption with corresponding total patient days for the year 2022. Data from hospitals in both systems were deduplicated using a trustee-based procedure, with ADKA-If-DGI data prioritised in case of overlap. 

The Table presents the number of the included non-university acute care hospitals and corresponding patient days, stratified by bed count, region, and ward group. The total number of patient days (PD) covered was 46,502,344 which represented around 50% of acute care hospital bed-days in Germany in 2022 [13]. After excluding the 35 university-affiliated hospitals, 525 non-university acute care hospitals (37,527,905 PD), covering over 45% of total bed-days nationwide for this hospital type, were included in the regional analyses. General wards accounted for 93% of all PDs for included non-university hospitals. 

**Table:** Characteristics of the included non-university acute care hospitals, Germany, 2022 (n = 525)

Characteristics	Number of hospitals (n = 525)	Total patient days (n = 37,527,905)
**Bed count**		
< 200	162	4,891,330
200–399	205	12,456,357
400–799	130	14,182,230
≥ 800	28	5,997,988
**Region**		
North-west	68	5,527,408
North-east	50	3,323,170
Centre/East	79	5,318,677
South-east	75	4,785,813
South-west	125	8,820,277
West	128	9,752,560
**Presence of general ward**		
Internal medicine (general)	501	13,866,144
Haematology/oncology	84	879,985
Other (non-surgical)	163	2,147,815
Surgery	486	9,142,510
Other (surgical/interdisciplinary)	433	9,003,055
**Presence of ICU**		
Non-surgical	122	554,007
Surgical/interdisciplinary	472	1,934,389

ICU: intensive care unit.

### Outcome measures

We included all drugs from Anatomical Therapeutic Chemical (ATC) group J01 (antibacterials for systemic use), as well as ATC codes P01AB01 (metronidazole – oral administration) and J04AB02 (rifampicin), that hospital pharmacies dispensed in 2022 to the inpatient wards. As detailed in Supplementary Table S2, we created the following subgroups of antibiotics for analysis: broad-spectrum penicillins (predominantly piperacillin/tazobactam), aminopenicillins with beta-lactamase inhibitors (aminopenicillins/BLI), narrow-spectrum penicillins, first- and second-generation cephalosporins, third- and fourth-generation cephalosporins, carbapenems, fluoroquinolones, glyco- and lipopeptides, aminoglycosides, macrolides/lincosamides, tetracyclines, antifolates/sulfonamides, linezolid and metronidazole.

We measured antibiotic use as AUD, based on the 2024 WHO ATC/DDD standard [14] and expressed it in WHO-DDD/100 PD. In the surveillance systems, patient days are calculated using real-time data where available; otherwise, the admission day is counted but the discharge day is not. To assess the robustness of the observed patterns, we additionally calculated AUD using hospital-adapted recommended daily doses (RDD/100 PD) [15] – an established alternative measure in the surveillance of hospital consumption in Germany. We evaluated AUD at the hospital level and aggregated it by ward type/admitting specialty, and also analysed it by route of administration (parenteral/oral). We also analysed the proportion of antibiotics from the WHO AWaRe categories.

### Statistical analyses

For descriptive analyses, we reported pooled DDD/100 PD per stratum as observed in the data. Where informative, we alternatively reported the observed median DDD/100 PD per hospital along with the interquartile range (IQR), defined by the upper and lower quartiles. The same measures were used for RDD/100 PD in sensitivity analyses.

We modelled regional differences in AUD using weighted mixed-effects zero-inflated Gamma regression per antibiotic group, with patient days as log-offset and data aggregated by hospital and at the ward type level described above [16]. We included ward type, hospital bed count, and region as fixed effects and added a hospital-level random effect to control for intra-hospital correlation of consumption. We used PD per unit as weights for the regression model to decrease the influence of smaller units with fewer patient days. West region (largest hospital count) served as the reference category, as did the category ‘Internal Medicine’ for ward type/admitting specialty and the category 200–399 beds for hospital size, respectively. For the probability of zero consumption we applied a simple intercept model. This approach mitigated potential confounding from differences in bed count and ward type distribution across regions. By combining specialty (e.g. internal, surgical) and care level (e.g. general, intensive care) into the ward type variable, it also controlled for variations in the proportion of intensive care beds.

To assess the statistical significance of differences in coefficients between regions we performed generalised linear hypothesis tests using the R-package glht with a Tukey multiple comparison correction per antibiotic group [17]. A corrected p value < 0.05 was defined as statistically significant.

As sensitivity analyses, we first performed negative binomial instead of zero-inflated Gamma regression with the same model structure. Second, we calculated standardised regional AUDs based on hospital size distribution and compared them to observed values, normalised by the overall pooled AUD. Differences in AUDs were considered more robust when appearing across all different types of analyses.

We performed all statistical analyses using R 4.2. For the mixed-effects zero-inflated Gamma and negative binomial regressions we used the R package glmmTMB [18].

## Results

### Overall consumption

The pooled AUD in the included non-university acute care hospitals was 51.8 DDD/100 PD, which was lower than in the participating university hospitals (66.8 DDD/100 PD), which, despite participating in the system, were excluded from the analysis. 

**Figure 2:**
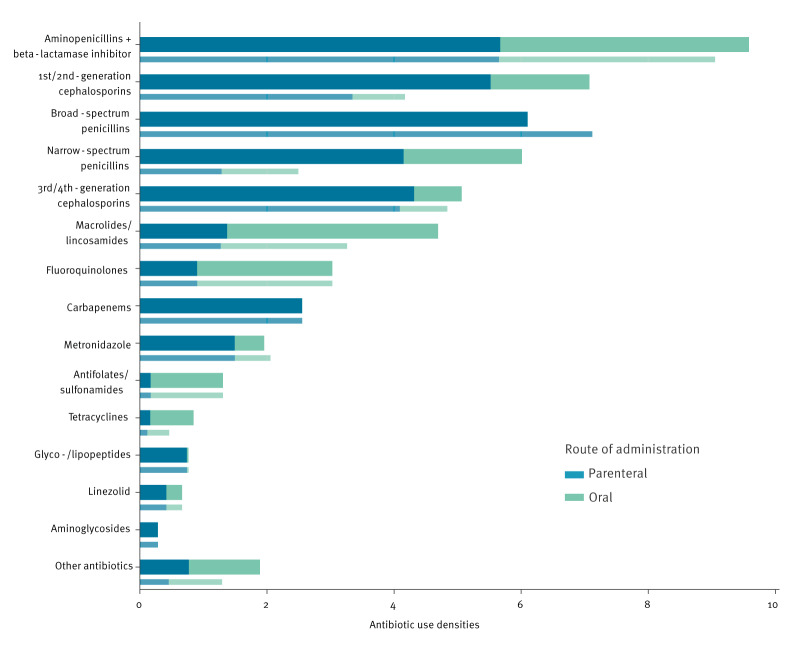
Antibiotic consumption densities for non-university acute care hospitals for different antibiotics and antibiotic classes, stratified by route of administration, Germany, 2022 (n = 525)

DDD: Defined Daily Doses; PD: patient days.

Figure 2 presents AUDs for the sample for different antibiotics and antibiotic classes, stratified by route of administration (parenteral/oral) for non-university acute care hospitals. Aminopenicillins/beta-lactamase inhibitors (BLIs) were the most frequently dispensed antibiotic group (9.6 DDD/100 PD; 18.5% of all DDDs) using either the DDD or RDD metric. Some differences emerged in certain beta-lactam groups when DDD or RDD served as the metric. In DDD/100 PD, first- and second-generation cephalosporins were the second most intensively used group (7.1 DDD/100 PD; 13.7%), followed by broad-spectrum (6.1 DDD/100 PD; 11.8%) and narrow-spectrum penicillins (6.0 DDD/100 PD; 11.6%). Third- and fourth-generation cephalosporins accounted for 9.8% of total consumption in DDD (5.1 DDD/100 PD), closely followed by macrolides with 9.1% (4.7 DDD/100 PD). Fluoroquinolones (3.0 DDD/100 PD; 5.8%) were used slightly more frequently than carbapenems (2.6 DDD/100 PD; 4.9%). Among all other antibiotics (14.9%), specific groups such as glyco- and lipopeptides, linezolid and aminoglycosides, each contributed less than 2% to total consumption. Details for the university hospitals on the consumption of the different drug classes are presented in Supplementary Figure S3.

Broad bar: antibiotic use densities in DDD/100 PD, slim bar: antibiotic use densities in RDD/100 PD.

### Analysis by bed count category

Non-university acute care hospitals with over 800 beds had the highest median overall AUD (54.9 DDD/100 PD; IQR: 50.4–60.0) (antibiotic consumption densities by hospital size and antibiotic group are detailed in Supplementary Table S3) and also the highest median AUDs for broad-spectrum penicillins, fluoroquinolones, carbapenems, antifolates/sulfonamides, linezolid, glyco- and lipopeptides and aminoglycosides. Hospitals with fewer than 200 beds showed the lowest median overall AUD (48.4 DDD/100 PD; IQR: 41.5–57.1) and the lowest median AUDs across most subgroups, except for aminopenicillins/BLI, where they had the highest AUD (10.3 DDD/100 PD; IQR: 6.5–13.1).

### Consumption by ward type and admitting specialty

Median AUD was considerably higher in ICUs (95.2 DDD/100 PD; IQR: 80.9–113.3) than in general wards (47.8 DDD/100 PD; IQR: 41.6–54.0), with surgical/interdisciplinary ICUs showing the highest value (97.3 DDD/100 PD; IQR: 83.0–119.1). Haematology departments also had increased antibiotic use (59.1 DDD/100 PD; IQR: 46.17–67.6) compared with the other internal medicine wards (47.3DDD/100 PD; IQR: 39.4–55.3). Intensive care units and haematology–oncology wards showed the highest carbapenem and linezolid use while fluoroquinolone use was highest in surgical wards, ICUs and haematology–oncology wards. Broad- and narrow-spectrum penicillin use followed similar patterns to the ones described for total antibiotic use. For additional results on antibiotic consumption densities by ward type, including quartiles and percentiles, see Supplementary Figure S4 and Supplementary Table S4.

### AWaRe (Access Watch Reserve) categories

In non-university acute care hospitals, antibiotics from the Access group accounted for 46.1% of total antibiotic consumption, with no discernible regional patterns. Use of Watch group antibiotics accounted for 51.4% of total antibiotic use, whereas those in the Reserve group accounted for 2.5%. Reserve group antibiotics showed slightly higher regional variation. Hospitals with more than 800 beds showed a higher proportion of Reserve antibiotic use (3.3%) compared with the overall average (2.5%), as well as a slightly lower proportion of Access antibiotic use (44.6% vs 46.1%). In regional comparison the highest share for Reserve use was in the NE region (3.6%), and the lowest in the NW region (2.5%). See Supplementary Figure S5 and Table S5 for antibiotic consumption by WHO-AWaRe category, stratified by region.

### Regional analyses for non-university acute care hospitals

**Figure 3:**
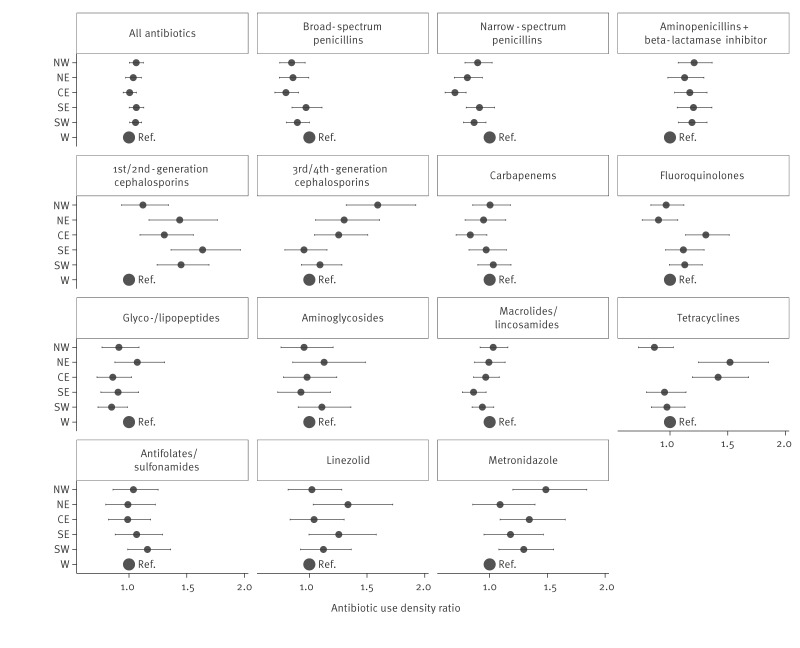
Regression analysis of regional antibiotic use, stratified by antibiotic group, Germany, 2022 (n = 525)

CE: Centre/East; NE: North-east; NW: North-west; SE: South-east; SW: South-west; W: West.

Figure 3 displays region-specific coefficients from the zero-inflated Gamma models stratified by antibiotic group. The Western region is used as the reference, and the model includes effects for ward type and bed count.

Representation of region-specific coefficients per region for the zero-inflated Gamma mixed-effects regressions incorporating the covariates region, bed count, and ward type for selected groups of antibiotics.

The full sets of model coefficients are shown in Supplementary Figures S10–S15 and Supplementary Tables S7–S12.

Using RDDs as an alternative consumption metric resulted in very similar coefficients (see Supplementary Figure S6 for comparison of regional effects in the regression model by outcome measure (DDD vs. RDD)). Supplementary Table S6 summarises observed and standardised antibiotic use densities by region. The probabilities of zero consumption for the model can be found in Supplementary Figure S7. The Supplementary materials also contain the comparison of the regression coefficients of the model with an alternative negative binomial regression model (see Supplementary Figure S8) and an overview of the observed and standardised regional AUDs (see Supplementary Figure S9) as described in the Methods section.

**Figure 4:**
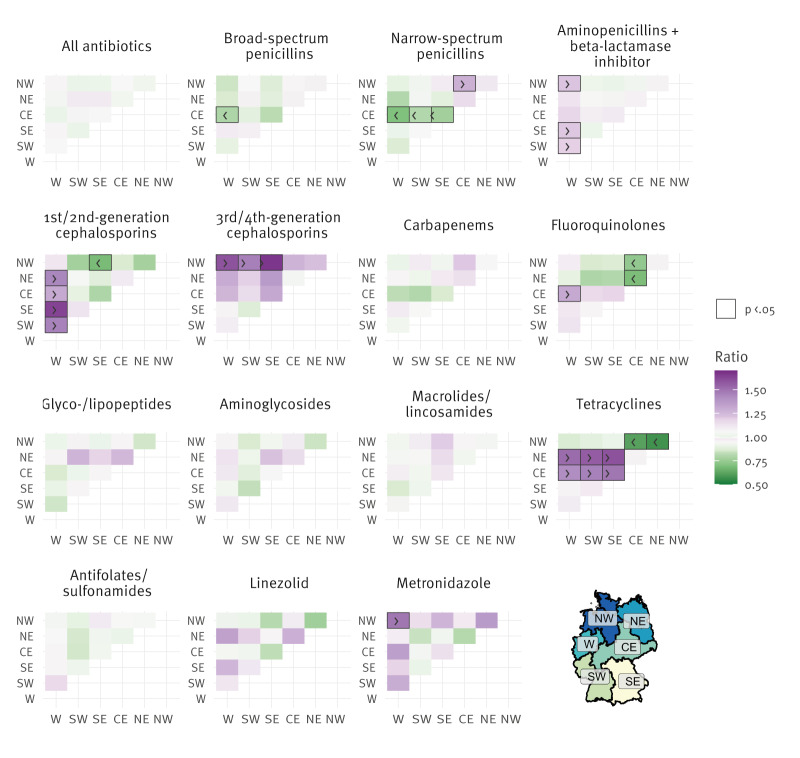
Heatmap of antibiotic use density ratios in non-university acute care hospitals between regions for the selected antibiotic groups, Germany, 2022 (n = 525)

CE: Centre/East; NE: North-east; NW: North-west; SE: South-east; SW: South-west; W: West.

Figure 4 displays a heatmap comparing AUD ratios between regions. Regional differences in antibiotic consumption were statistically significant for a limited number of antibiotic groups and emerged consistently across all types of analyses.

Statistically significant differences are marked with a black border (p < 0.05). Direction of the effect is indicated by >/<  signs comparing the Y-axis with the X-axis. The regression models are based on data from 525 German non-university acute care hospitals. The map shows the considered regions, with different colours used for better visualisation.

### Beta-lactam antibiotics

Within the group of beta-lactam antibiotics, we observed regional differences across almost all subgroups. Broad-spectrum penicillins showed relatively uniform use except for significant differences between the CE and the W region. Aminopenicillin/BLI combinations were most heavily used in NW, SE, and SW compared with the W region. Narrow-spectrum penicillin use was notably lower in CE compared with other regions, particularly NW, W, SE and SW. Use of first- and second -generation cephalosporins was more frequent in SE and also significantly higher in NE, CE, and SW compared with W. The group of third- and fourth-generation cephalosporins showed the highest consumption ratios in NW with SE recording the lowest. In contrast, carbapenem use was relatively consistent across regions, with only slightly lower levels in CE and NW and no statistically significant differences overall.

### Fluoroquinolones

The highest AUD of fluoroquinolones were modelled in CE, which was significantly higher compared with the W region. In contrast, the lowest usage was modelled in NW and NE.

### Other antibiotics

Tetracyclines – among the least used antibiotics overall – showed greater regional differences, with the highest modelled AUDs in NE and CE, and which were statistically significantly higher than in all other regions. Metronidazole consumption showed some variations across regions and was modelled highest in NW, compared with the lowest use in W and NE. Although NE and W were modelled to have higher AUDs for glyco- and lipopeptides, no statistically significant differences were observed. Linezolid consumption did not show any significant differences.

## Discussion

In response to the public health threat posed by AMR, AMR and antibiotic stewardship are increasingly prioritised in healthcare policy, highlighting the need for detailed analyses of antibiotic consumption patterns. Germany lacks a fully comprehensive nationwide antibiotic consumption monitoring system. Population-based estimates have enabled inclusion of national hospital surveillance data in the European Surveillance of Antimicrobial Consumption Network (ESAC-Net) for European comparisons only since 2023 [19].

This study provides a large-scale regional analysis of hospital antibiotic use in Germany, covering over 45% of non-university acute care bed-days. We observed statistically significant regional variations, influenced by hospital size, departmental structure and geographic location. These patterns were confirmed through robust regression and sensitivity analyses, including the use of the RDD metric. Overall consumption aligns with patterns seen in Northern and Central Europe [20], though notable differences emerged in specific antibiotic classes. Smaller hospitals and general wards, despite lower per-patient use, contribute substantially to national antibiotic consumption. Our findings support targeted antibiotic stewardship and present a methodological approach for detecting subnational prescribing differences, potentially serving as a model for countries with similar surveillance coverage. With an overall antibiotic use of 51.8 DDD per 100 PD in our sample, Germany would be positioned in the lower third among European countries, alongside Austria, Norway and Poland. Although Germany was not part of the European hospital antibiotic consumption study (2024, data from 2017–21) [20], our data align with their findings, indicating lower use compared with countries like Spain, Italy and Czechia. Descriptive analysis of antibiotic consumption revealed that in 2022, aminopenicillins/BLI were the most commonly prescribed antibiotics in our sample, followed by first- and second-generation cephalosporins.

Antibiotic use increased with the hospital size for most antibiotic groups and specifically for WHO Reserve group antibiotics. This was anticipated, as larger hospitals often have more complex case-mix and higher patient turnover [21]. Smaller hospitals (≤ 399 beds) showed lower overall antibiotic use but slightly higher macrolide and tetracycline consumption, which may reflect relatively more community-acquired infections and fewer complicated infections. Although individual use is lower, smaller hospitals’ cumulative impact is notable, underscoring the need for tailored antimicrobial stewardship, especially in facilities lacking multidisciplinary antimicrobial stewardship (AMS) teams [22]. In Germany, AMS teams follow national guidelines, but impact of their measures on antibiotic use varies depending on staffing and clinical integration.

At the ward type and admitting specialty level, ICUs in general and haematology–oncology wards had the highest AUDs, while general wards, especially internal medicine, accounted for the largest absolute use because of patient volume. This aligns with previous studies showing that while ICUs have higher consumption per patient, general wards drive total antibiotic use in absolute terms [23]. This confirms that both high-density and high-volume areas should be targets for optimisation.

The WHO AWaRe classification divides antibiotics into Access, Watch and Reserve groups to support benchmarking. Although this classification is not specifically adapted to the German context and has limitations for detailed national analysis, it still serves as a useful tool for assessing antibiotic use. In our analysis, Access group antibiotics made up 46.1% of overall consumption, with no clearly visible regional variation. Combined Access group antibiotic consumption from community and hospital sector was estimated to be around 60% in 2023 in the most recent European Centre for Disease Prevention and Control (ECDC) ESAC-Net surveillance report [19]. 

An important finding of the present work was that the overall hospital antibiotic use appeared regionally balanced. This was unexpected, as outpatient prescribing in Germany has shown significant and persistent regional variation [5], and earlier studies exploring the relationship between regional inpatient and outpatient antibiotic prescribing, for example in Italy [7] or Hungary [24] suggest that low use in one sector may be compensated by higher use in another. While overall use variation was limited, notable differences emerged when analysing antibiotic classes. Statistically significant variation occurred in various beta-lactam antibiotic classes, tetracyclines and fluoroquinolones.

Higher use of third- and fourth-generation cephalosporins in the NW correlated with elevated metronidazole use, underscoring the need for granular bedside investigations, such as point prevalence studies, to better understand prescribing rationales. This is particularly relevant for *Clostridioides difficile* infections and varying prescriber awareness. A recent German study reported redundant anaerobic therapy most frequently as penicillin/BLI plus metronidazole (38%), penicillin/BLI plus clindamycin (17%) and carbapenem plus metronidazole (15%) [25]. Higher consumption of first- and second-generation cephalosporins in many regions, especially in surgical departments, is difficult to explain without further detailed prescribing analysis. Possible factors include prolonged perioperative prophylaxis or a rising number of surgical procedures, many of which required antibiotic prophylaxis. While the proportion of treated patients likely remains stable, declining length of stay suggests that expressing use as DDD per 100 admissions may better capture consumption trends over time. The pattern may also indicate regional variation in the implementation or effectiveness of antimicrobial stewardship efforts including their impact on reducing prolonged perioperative prophylaxis [26]. Tetracyclines, among the least used antibiotics overall, show higher usage in the NE and CE regions, which is noteworthy and requires in depth analysis regarding the main indications and the most frequent specific drugs. Although the nationwide overall fluoroquinolone consumption remained low, in line with lower consumption reported by ESAC-Net [19] in recent years for the whole EU, we observed high use in one of the regions which, again, highlights the importance of targeted local antimicrobial stewardship strategies to address regional overuse and ensure safe, guideline-concordant prescribing practices. No major regional variation was detected for other antibiotic classes including carbapenems, glyco- and lipopeptides and linezolid.

A key strength of this study is its large-scale, nationwide dataset, offering a comprehensive overview of hospital antibiotic consumption patterns in Germany. Recommended daily doses (RDD), representing the recommended daily drug dose in the German context and aligning with treatment guidelines, were applied as a complementary sensitivity measure. Due to differences between DDD and RDD, e.g. lower DDDs for several beta-lactams lead to an overestimation of AUDs, overall antibiotic use showed expected variations across and within antibiotic groups. Nonetheless, patterns in the regression models were largely reproduced and confirmed. The robustness of the observed patterns was supported by complementary methods, including observed and standardised AUDs, allowing regional comparisons across hospitals. Additionally, regression models were applied to ward-level data, adjusting for confounders such as bed count and ward type (including department specialty and level of care). While observed and standardised aggregate measures may reflect biases caused by study population composition — since they did not adjust for ward type — the regression models were sensitive to the underlying statistical assumptions. By combining these different sensitivity analyses, we strengthened the reliability and validity of our findings.

Similar to our analysis, a previous United States-based study [8] used regression models to assess regional differences in antibiotic use, relying on hospital-level data with available case-mix information. In contrast, our ward-level approach incorporated hospital-level random effects and demonstrated how missing case-mix data can be at least partially offset by detailed ward-type information including level of care. As information on ward type and level of care is sometimes easier to obtain than case-mix indicators, comparing both methods using the same hospital sample could be a valuable direction for future research.

This study has several limitations, including reliance on aggregated data, which prevents analysis of individual patient factors and clinical appropriateness of antibiotic use. Regional variations in infection patterns, diagnostic practices and clinical decision making are not captured which limits the interpretation of the presented results. Excluding university hospitals may limit the analysis, but focusing on non-university hospitals helps clarify regional patterns, as university hospitals’ educational role and distinct patient mix may be less affected by regional factors beyond local pathogen and resistance prevalence [21]. University hospitals, because of their academic interconnectedness, may develop similar treatment practices, which could reduce visible regional differences. Additionally, the 2022 antibiotic consumption data reflect the post-COVID-19 pandemic period, when changes in healthcare practices, patient behaviour, and infection rates likely influenced usage patterns [27,28]. These shifts emphasise the need for cautious interpretation of the results.

## Conclusions

This study reveals regional differences in antibiotic use across Germany, particularly for beta-lactams, tetracyclines and fluoroquinolones. These patterns are likely shaped by a mix of epidemiological, institutional and educational factors. Elucidating these influences is key for future research. Targeted interventions should focus on high-use settings, especially where broad-spectrum or reserve antibiotics are overused. Prospective research should serve to improve communication of therapeutic standards and strengthen antimicrobial stewardship regionally and within hospitals.

## Data Availability

Aggregated data sets are available upon reasonable request from the corresponding author.

## Supplementary Data

2243000 Supplement
