# Supplementary material for: Antibiotic consumption patterns in acute care hospitals: an integrated analysis using regression modelling combining data from two surveillance systems, Germany, 2022
Source: Euro Surveill. 2025 Nov 13;30(45):2500560. doi: 10.2807/1560-7917.ES.2025.30.45.2500560 (PMC12633710; doi:10.2807/1560-7917.ES.2025.30.45.2500560)
Supplement: Supplement [file 25-00560_GROESCHNER_Supplement.pdf]

This supplementary material is hosted by *Eurosurveillance* as supporting information alongside the article [**Antibiotic Consumption Patterns in German Acute Care Hospitals in 2022: An Integrated Analysis Using Regression Modelling Combining Data from Two Surveillance Systems**], on behalf of the authors, who remain responsible for the accuracy and appropriateness of the content. The same standards for ethics, copyright, attributions and permissions as for the article apply. Supplements are not edited by *Eurosurveillance* and the journal is not responsible for the maintenance of any links or email addresses provided therein.

Supplementary material

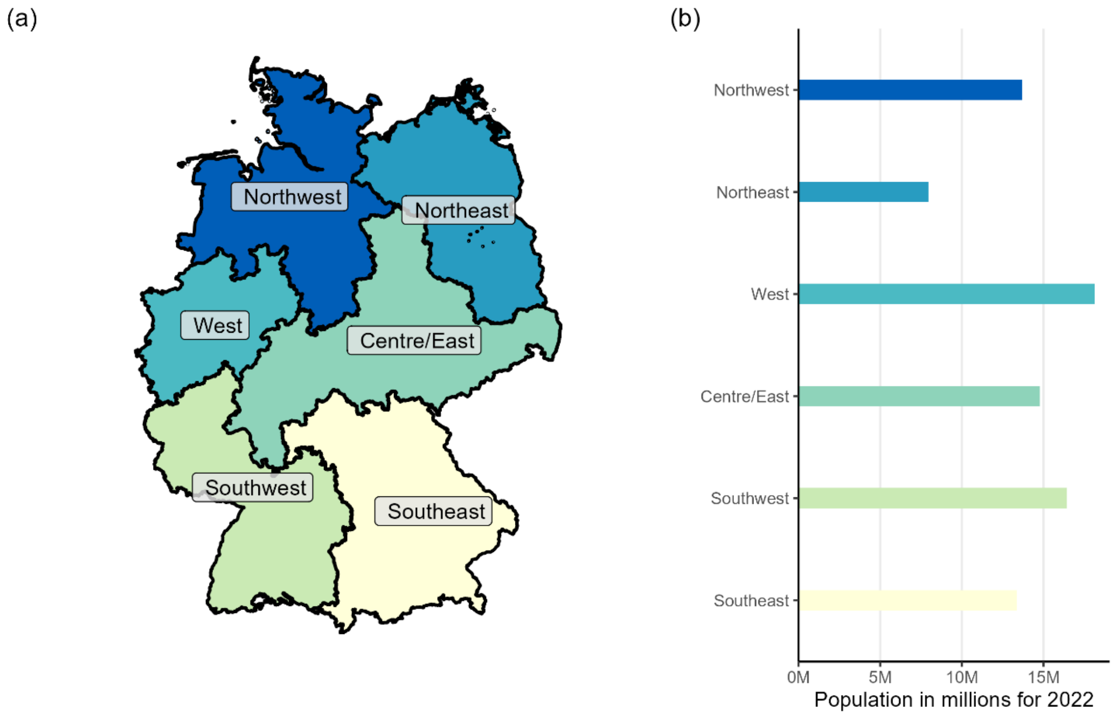

Figure S1: *Definition of regions used for analyses of antibiotic consumption in non-university hospitals (a) and population numbers for these regions (b).*
Northwest: Hanseatic City of Hamburg, Lower Saxony, Hanseatic City of Bremen, Schleswig-Holstein; Northeast: Berlin, Brandenburg, Mecklenburg-Western Pomerania; West: North Rhine-Westphalia; Centre/East: Saxony, Saxony-Anhalt, Thuringia, Hesse; Southwest: Baden-Wurttemberg, Saarland, Rhineland-Palatinate, Southeast: Bavaria

**General wards**	
**Medical wards**	
Internal Medicine	*General internal medicine, gastroenterology, cardiology, endocrinology, nephrology, infectiology, pneumology, rheumatology, geriatrics, palliative medicine, COVID unit*
	Haematology/Oncology
Other (non-surgical)	*Neurology, radiology, nuclear medicine*
**Surgical wards**	
Surgery	*General and visceral surgery, vascular surgery, cardiothoracic surgery, plastic surgery, hand surgery, burn surgery, neurosurgery, trauma surgery/ orthopaedics, spinal surgery, transplant surgery*
Other (surgical/interdisciplinary)	*Ophthalmology, dermatology, urology, maxillofacial surgery, gynaecology/obstetrics*
**ICU**	
Non-Surgical	*Cardiology, angiology, internal medicine, gastroenterology, pneumology, hepatology, neurology*
Surgical/Interdisciplinary	*surgical and interdisciplinary ICU wards*

***Table S1**: Definition of ward groups based on ward type and department.*

Abbreviations: COVID - corona virus disease, ICU - intensive care unit

Antibiotic/ antibiotic group	ATC-Codes
**All antibiotics**	J01 (antibacterials for systemic use) / P01AB01 (metronidazole) / J04AB02 (rifampicin)
**Broad-spectrum penicillins**	J01CA09 (azlocillin) / J01CA10 (mezlocillin) / J01CA12 (piperacillin) / J01CA17 (temocillin) / J01CR03 (ticarcillin/BLI) / J01CR05 (piperacillin/BLI)
**Narrow-spectrum penicillins**	J01CA01 (ampicillin)/ J01CA04 (amoxicillin) / J01CA08 (pivmecillinam) / J01CE (β-lactamase sensitive penicillins) / J01CF (β-lactamase resistant penicillins)
**Aminopenicillins/ß-lactamase inhibitor**	J01CR01 (ampicillin/BLI) / J01CR02 (amoxicillin/BLI) / J01CR04 (sultamicillin)
**1./2. generation cephalosporins**	J01DB (1st generation cephalosporins) / J01DC (2nd generation cephalosporins)
**3./4. generation cephalosporins**	J01DD (3rd generation cephalosporins) / J01DE01 (cefepim) / J01DI04 (cefiderocol)
**Carbapenems**	J01DH
**Fluoroquinolones**	J01MA
**Glyco- and lipopeptides**	J01XA
**Aminoglycosides**	J01GA (streptomycin), J01GB01 (tobramycin), J01GB03 (gentamicin), J01GB06 (amikacin)
**Macrolides/lincosamides**	J01FA (macrolides) / J01FF (lincosamides)
**Tetracyclines**	J01AA01 (demedocycline), J01AA02 (doxycycline), J01AA07 (tetracycline), J01AA08 (minocycline), J01 (tigecycline)
**Antifolates/sulfonamides**	J01EA01 (trimethoprim), J01EC01 (sulfamethoxazole), J01EC02 (sulfadiazine)
**Linezolid**	J01XX08
**Metronidazole**	P01AB01 (oral) / J01XD01 (parenteral)
**Other antibiotics**	All other antibiotics not included in one of the subgroups above

Table S2: *Definition of antibiotic groups based on the corresponding ATC-Codes. See https://atcddd.fhi.no/atc_ddd_index/ for precise definition of the ATC-Codes.*

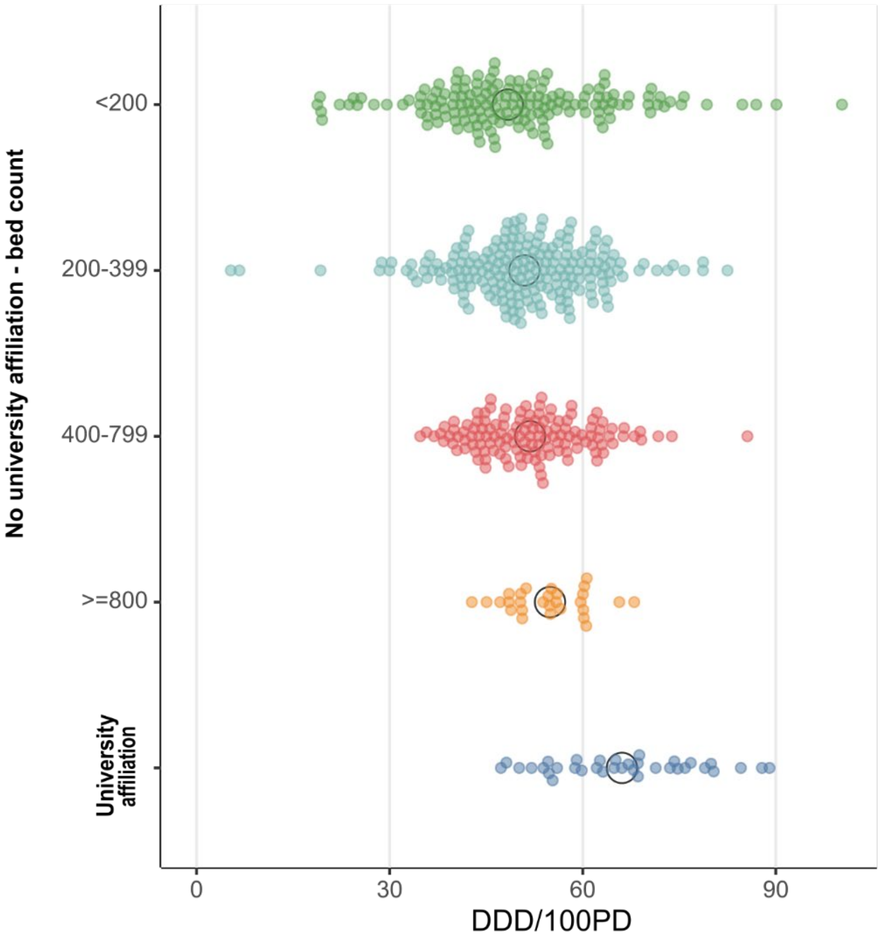

Figure S2: *Antibiotic use densities (AUD) for all antibiotics in DDD/100PD for the whole study population of 525 non-university acute care hospitals and 35 university hospitals. Non-university hospitals are stratified by bed count. Individual hospital AUDs are represented by coloured dots. The large black circles show the median AUD per category.*

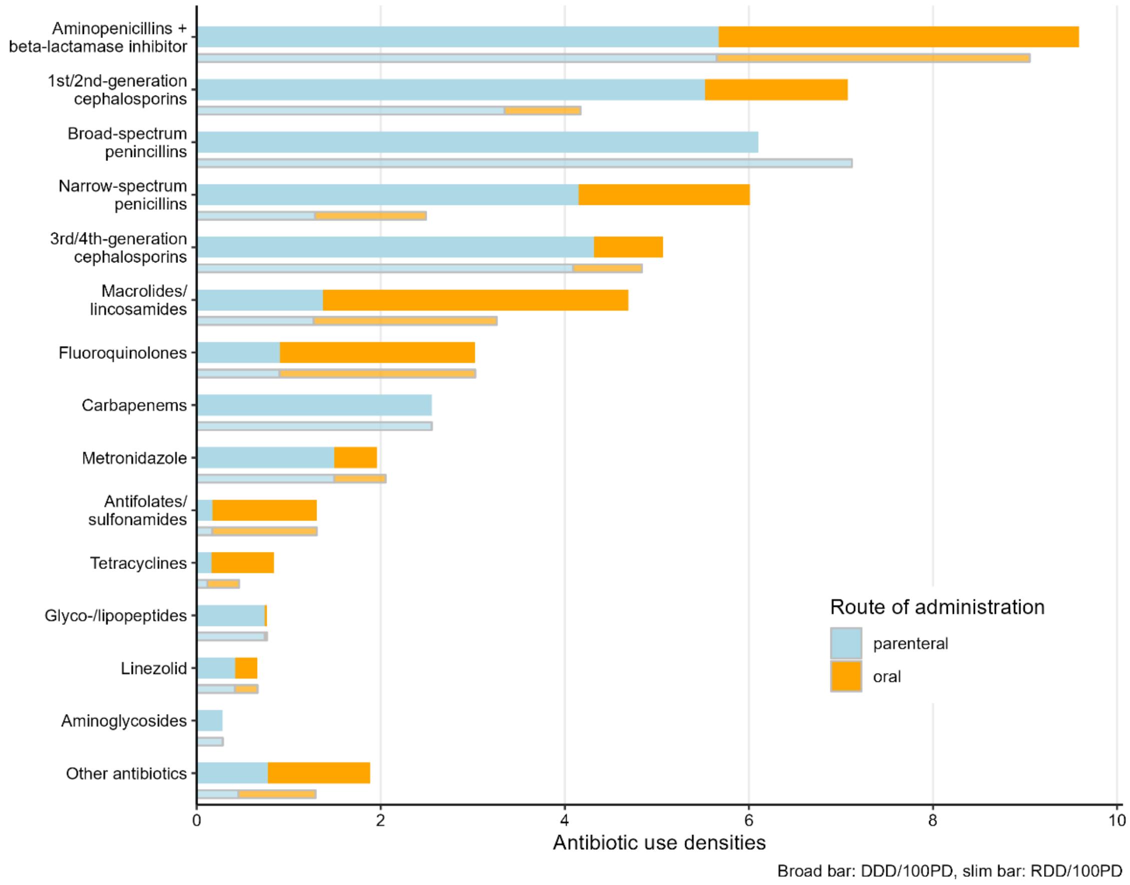

Figure S3: *Pooled antibiotic use densities for 2022 of antibiotic groups by route of administration (parenteral/ oral) for 35* ***university hospitals*** *in study population expressed in DDD/100PD (broad bar) and RDD /100 PD (slim bar).*

Consumption in DDD/100PD - Median (Lower quartile, Upper quartile)				
	<200	200-399	400-799	≥800
**All antibiotics**	**48.36 (41.5, 57.14)**	**50.93 (45.46, 57.69)**	**51.81 (45.6, 57.55)**	**54.92 (50.36, 60.04)**
Broad-spectrum penicillins	5.3 (3.57, 7)	5.96 (4.57, 7.1)	6.23 (5.05, 7.51)	6.56 (5.66, 7.43)
Narrow-spectrum penicillins	4.71 (3.03, 6.64)	5.8 (4.06, 7.66)	5.78 (4.74, 7.32)	5.77 (4.33, 7.27)
Aminopenicillins/ β-lactamase inhibitor	10.28 (6.51, 13.09)	9.46 (7.09, 12.07)	8.47 (6.98, 10.87)	9.39 (7.65, 11.25)
1./2. generation cephalosporins	5.73 (2.85, 8.98)	6.38 (3.73, 9.3)	6.5 (4.27, 8.93)	6.66 (5.37, 8.27)
3./4. generation cephalosporins	3.85 (2.07, 6.41)	4.38 (2.52, 6.38)	5.2 (2.89, 7.58)	4.34 (3.36, 5.69)
Carbapenems	1.7 (1.01, 2.55)	1.91 (1.37, 2.86)	2.38 (1.88, 3.38)	3.01 (2.43, 3.47)
Fluoroquinolones	2.72 (1.65, 3.78)	2.59 (1.71, 3.56)	2.67 (2.15, 3.46)	3.53 (3.08, 4.44)
Glyco-/lipopeptides	0.48 (0.34, 0.74)	0.5 (0.34, 0.74)	0.67 (0.46, 0.98)	0.84 (0.65, 1.19)
Aminoglycosides	0.14 (0.06, 0.26)	0.19 (0.11, 0.3)	0.23 (0.13, 0.39)	0.29 (0.24, 0.42)
Macrolides/lincosamides	4.41 (3.29, 6.3)	4.91 (3.61, 6.13)	4.43 (3.59, 5.43)	3.95 (3.52, 4.87)
Tetracyclines	0.59 (0.33, 1.03)	0.65 (0.45, 0.99)	0.69 (0.48, 1.04)	0.92 (0.64, 1.27)
Antifolates/sulfonamides	0.79 (0.48, 1.47)	0.83 (0.52, 1.38)	1.12 (0.68, 1.8)	1.48 (1.16, 1.86)
Linezolid	0.28 (0.12, 0.6)	0.47 (0.26, 0.76)	0.55 (0.36, 0.87)	0.68 (0.52, 1.05)
Metronidazole	1.62 (0.59, 2.98)	1.88 (1.13, 2.81)	1.68 (1.19, 2.47)	1.91 (0.96, 2.28)

Table S3: *Antibiotic use densities in DDD/100PD for different hospital sizes and antibiotic groups in 525 non-university acute care hospitals.*

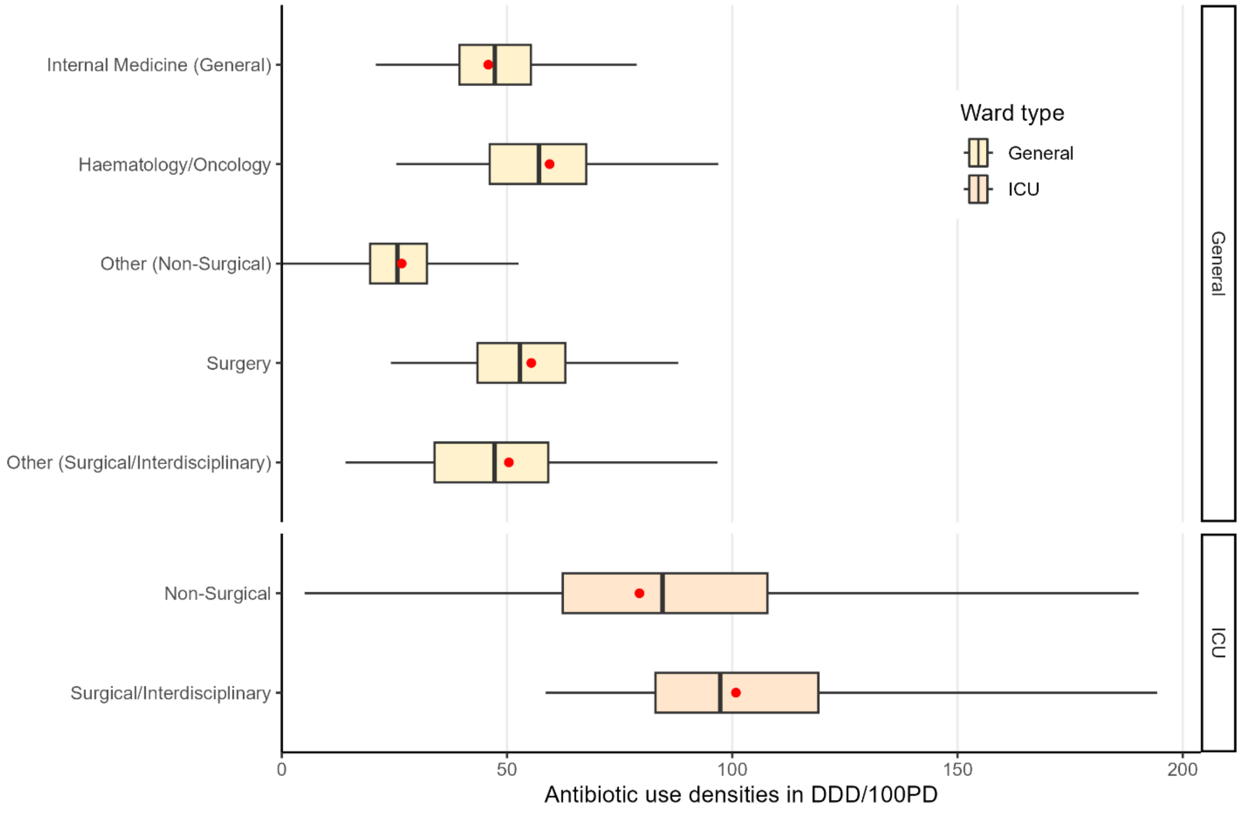

***Figure S4**: Antibiotic use densities in DDD/100PD shown on ward type/ admitting specialty level. Following standard convention, the rectangles and the bold vertical lines represent lower quartile, median and upper quartile. Whiskers of the bar plot show 2.5- and 97.5- percentiles. The red dots show pooled antibiotic use densities in the 525 non-university acute care hospitals.*

Consumption in DDD/100PD - Median (Lower quartile, Upper quartile)									
	**General ward**						**ICU**		
	**All**	Internal Medicine (General)	Haematology/Oncology	Other (Non-Surgical)	Surgery	Other (Surgical/Interdisciplinary)	**All**	Non-Surgical	Surgical/Interdisciplinary
**All antibiotics**	**47.79 (41.6, 53.97)**	**47.3 (39.43, 55.3)**	**57.09 (46.17, 67.6)**	**25.67 (19.6, 32.23)**	**52.89 (43.46, 62.95)**	**47.23 (33.92, 59.16)**	**95.2 (80.88, 113.33)**	**84.53 (62.38, 107.8)**	**97.34 (82.95, 119.11)**
Broad-spec. pen.	5.01 (3.71, 6.28)	6.05 (4.44, 8.04)	11.4 (9.08, 13.9)	3.56 (1.94, 5.07)	3.95 (2.32, 5.57)	3.45 (1.7, 5.47)	18.64 (14.96, 22.73)	14.95 (10.22, 21.87)	19.23 (15.4, 23.22)
Narrow-spec. pen.	5.28 (3.8, 7.09)	5.89 (4.04, 8.44)	3.96 (2.55, 6.6)	3.45 (2.23, 4.82)	4.51 (2.55, 7.1)	4.29 (2.79, 6.09)	9.34 (5.48, 14.39)	7.71 (4.03, 14.18)	9.59 (5.72, 14.74)
Aminopen. /BLI	9.32 (7.04, 12.5)	10.32 (7.49, 13.42)	6.19 (4.17, 8.99)	4.13 (2.63, 6.09)	9.31 (6.12, 12.72)	8.08 (4.36, 13.15)	7.9 (5.35, 10.92)	7.75 (5, 12.44)	7.66 (5.15, 10.79)
1./2. gen. ceph.	6.19 (3.83, 9.22)	1.69 (0.94, 2.76)	1.19 (0.66, 1.76)	0.94 (0.44, 1.7)	12.16 (7.92, 18.14)	7.07 (4.04, 11.99)	4.71 (2.62, 8.63)	2.52 (1.07, 4.21)	5.2 (2.81, 9.21)
3./4. gen. ceph.	4.31 (2.39, 6.5)	4.82 (2.74, 7.26)	3.89 (2.6, 5.86)	3.83 (2.4, 5.85)	2.52 (1.34, 4.7)	3.59 (1.56, 7.26)	6.22 (4.11, 9.12)	6.62 (3.67, 9.19)	6.29 (4.14, 9.31)
Carbapenems	1.4 (0.93, 2.09)	1.66 (1.04, 2.42)	6.3 (3.51, 7.87)	0.83 (0.37, 1.4)	1.05 (0.61, 1.86)	0.98 (0.37, 1.93)	11.66 (8.19, 17.16)	9.53 (4.76, 13.81)	12.17 (8.49, 17.74)
Fluoroquinolones	2.5 (1.78, 3.46)	2.58 (1.66, 3.73)	4.56 (2.56, 7.88)	0.88 (0.36, 1.78)	2.35 (1.59, 3.42)	2.08 (0.98, 3.41)	4.85 (2.8, 7.89)	5.07 (2.96, 8.29)	4.66 (2.56, 7.6)
Glyco-/lipopeptides	0.48 (0.31, 0.67)	0.46 (0.29, 0.66)	1.07 (0.67, 2.04)	0.23 (0.09, 0.53)	0.5 (0.23, 0.84)	0.26 (0.1, 0.52)	1.76 (0.89, 3.16)	1.46 (0.57, 2.8)	1.83 (0.92, 3.44)
Aminoglycosides	0.14 (0.07, 0.24)	0.14 (0.05, 0.28)	0.1 (0, 0.24)	0.07 (0, 0.19)	0.08 (0.01, 0.2)	0.09 (0.01, 0.25)	0.73 (0.34, 1.43)	0.59 (0.22, 1.27)	0.73 (0.38, 1.54)

Consumption in DDD/100PD - Median (Lower quartile, Upper quartile)									
	**General ward**						**ICU**		
	**All**	Internal Medicine (General)	Haematol-ogy/Oncol-ogy	Other (Non-Surgical)	Surgery	Other (Surgi-cal/Interdisci-plinary)	**All**	Non-Surgical	Surgical/Inter-disciplinary
Macrolides/lin-cos.	4.22 (3.2, 5.63)	4.93 (3.72, 7.13)	4.46 (2.9, 5.58)	1.35 (0.81, 2.19)	3.51 (2.36, 4.96)	3.38 (1.83, 5.05)	9.57 (6.72, 13.31)	8.54 (4.76, 12.16)	9.63 (6.7, 13.4)
Tetracyclines	0.62 (0.39, 0.96)	0.45 (0.22, 0.78)	0.87 (0.33, 1.55)	0.49 (0.17, 0.89)	0.47 (0.2, 0.91)	0.86 (0.44, 1.42)	0.77 (0.18, 1.77)	0.71 (0, 1.61)	0.74 (0.17, 1.84)
Antifol. /sul-fonamides	0.89 (0.53, 1.47)	0.72 (0.41, 1.16)	3.58 (2.08, 6.63)	0.66 (0.31, 1.26)	0.73 (0.39, 1.32)	0.71 (0.32, 1.59)	1.19 (0.54, 2.32)	1.38 (0.52, 2.76)	1.15 (0.49, 2.28)
Linezolid	0.3 (0.15, 0.56)	0.27 (0.12, 0.51)	1.09 (0.27, 2.04)	0.06 (0, 0.2)	0.33 (0.14, 0.7)	0.15 (0.03, 0.36)	2.55 (1.14, 4.52)	1.49 (0.32, 2.87)	2.87 (1.22, 4.9)
Metronidazole	1.73 (0.97, 2.67)	1.21 (0.61, 2.07)	1.4 (0.71, 2.09)	0.37 (0.18, 0.82)	2.41 (1.02, 4.45)	1.65 (0.85, 2.55)	2.22 (1.11, 4.13)	0.92 (0.44, 1.64)	2.44 (1.29, 4.46)

Table S4: *Antibiotic use densities in DDD/100PD (median and IQR) for ward type/ admitting specialty and antibiotic groups in 525 non-university acute care hospitals.*

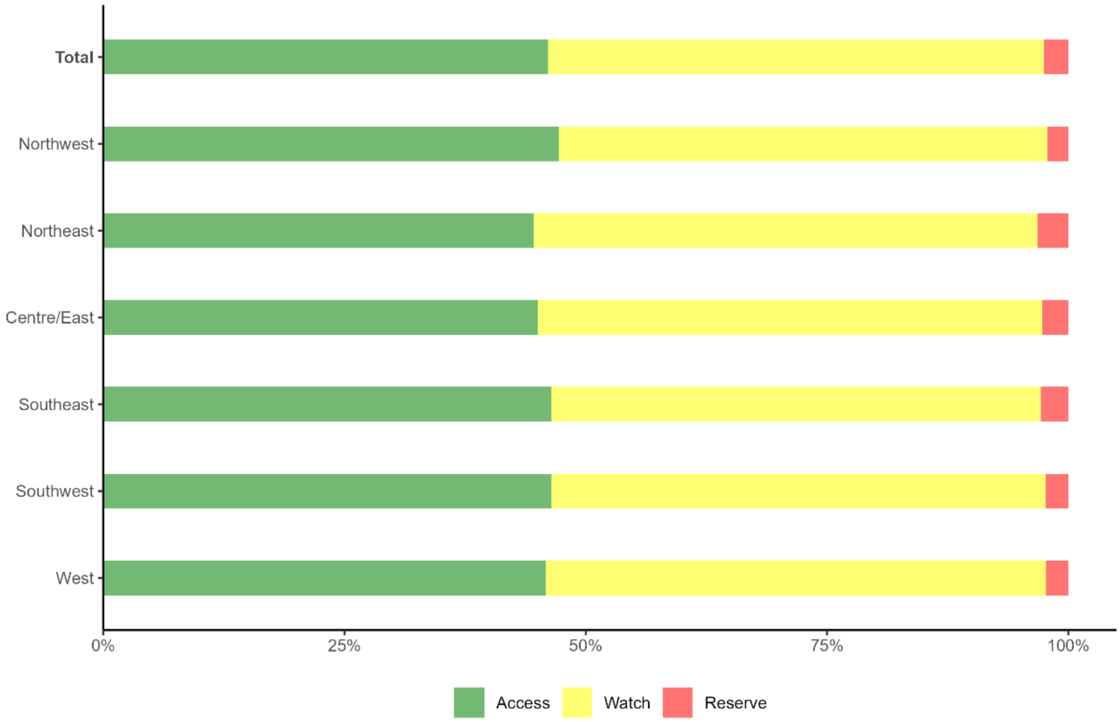

***Figure S5**: Proportion of antibiotic use in DDD by WHO-AWaRe-category stratified by region and for the whole sample (Total).*

**Bed count**	**Access**	**Watch**	**Reserve**
**All**	46.1%	51.4%	2.5%
**<200**	46,56%	51,34%	2,09%
**200-399**	46,76%	50,97%	2,27%
**400-799**	45,92%	51,59%	2,49%
**≥800**	44,63%	52,04%	3,33%

***Table S5**: Proportion of antibiotic use in DDD by WHO-AWaRe-category stratified by bed count category for the whole sample (Total).*

		Consumption in DDD/100PD (*RDD/100PD*)					
		Northwest	Northeast	Centre/East	Southeast	Southwest	West
**All antibiotics**	**Obs.:**<br>**Std.:**	**53.01 (*44.83*)**<br>**52.67 (*44.47*)**	**51.93 (*43.15*)**<br>**51.63 (*42.9*)**	**50.2 (*42.44*)**<br>**49.71 (*42.04*)**	**54.11 (*44.76*)**<br>**54.2 (*44.77*)**	**53.12 (*44.29*)**<br>**53.12 (*44.28*)**	**49.68 (*41.48*)**<br>**49.81 (*41.62*)**
Broad-spectrum penicillins	Obs.:<br>Std.:	5.6 (*6.53*)<br>5.52 (*6.45*)	5.6 (*6.54*)<br>5.58 (*6.51*)	5.58 (*6.51*)<br>5.64 (*6.58*)	6.87 (*8.02*)<br>6.89 (*8.04*)	6.11 (*7.13*)<br>6.12 (*7.14*)	6.46 (*7.54*)<br>6.47 (*7.55*)
Narrow-spectrum penicillins	Obs.:<br>Std.:	5.92 (*2.55*)<br>6.11 (*2.65*)	5.66 (*2.36*)<br>5.64 (*2.36*)	5.03 (*2.1*)<br>5.11 (*2.17*)	6.55 (*2.61*)<br>6.8 (*2.7*)	5.9 (*2.34*)<br>5.91 (*2.34*)	6.55 (*2.8*)<br>6.49 (*2.78*)
Aminopenicillins + beta-lactamase inhibitor	Obs.:<br>Std.:	10.17 (*9.56*)<br>10.13 (*9.54*)	9.93 (*9.4*)<br>9.8 (*9.28*)	9.76 (*9.18*)<br>9.72 (*9.13*)	10.07 (*9.5*)<br>10.11 (*9.52*)	10.17 (*9.66*)<br>10.14 (*9.63*)	8.27 (*7.81*)<br>8.33 (*7.87*)
1st/2nd-generation cephalosporins	Obs.:<br>Std.:	6.68 (*3.96*)<br>6.56 (*3.88*)	7.2 (*4.3*)<br>7.19 (*4.29*)	6.28 (*3.72*)<br>6.16 (*3.65*)	8.69 (*5.13*)<br>8.39 (*4.94*)	7.99 (*4.67*)<br>8.01 (*4.69*)	6.06 (*3.56*)<br>6.05 (*3.57*)
3rd/4th-generation cephalosporins	Obs.:<br>Std.:	6.52 (*6.33*)<br>6.41 (*6.23*)	5.45 (*5.24*)<br>5.46 (*5.25*)	5.34 (*5.05*)<br>5.33 (*5.05*)	4.05 (*3.81*)<br>4.22 (*3.94*)	4.76 (*4.49*)<br>4.76 (*4.49*)	4.73 (*4.56*)<br>4.64 (*4.47*)
Carbapenems	Obs.:<br>Std.:	2.5 (*2.5*)<br>2.46 (*2.46*)	2.56 (*2.56*)<br>2.5 (*2.5*)	2.36 (*2.36*)<br>2.27 (*2.27*)	2.77 (*2.77*)<br>2.73 (*2.73*)	2.58 (*2.58*)<br>2.58 (*2.58*)	2.56 (*2.56*)<br>2.55 (*2.55*)
Fluoroquinolones	Obs.:<br>Std.:	2.83 (*2.83*)<br>2.84 (*2.84*)	2.46 (*2.46*)<br>2.46 (*2.46*)	3.48 (*3.48*)<br>3.36 (*3.36*)	3.12 (*3.12*)<br>3.13 (*3.13*)	3.28 (*3.28*)<br>3.27 (*3.27*)	2.81 (*2.81*)<br>2.9 (*2.9*)
Glyco-/lipopeptides	Obs.:<br>Std.:	0.84 (*0.84*)<br>0.81 (*0.81*)	0.98 (*0.98*)<br>0.96 (*0.96*)	0.68 (*0.68*)<br>0.67 (*0.67*)	0.79 (*0.79*)<br>0.78 (*0.79*)	0.64 (*0.64*)<br>0.64 (*0.64*)	0.79 (*0.79*)<br>0.8 (*0.8*)
Aminoglycosides	Obs.:<br>Std.:	0.25 (*0.25*)<br>0.26 (*0.26*)	0.29 (*0.29*)<br>0.29 (*0.29*)	0.31 (*0.31*)<br>0.29 (*0.29*)	0.23 (*0.23*)<br>0.24 (*0.24*)	0.29 (*0.29*)<br>0.28 (*0.28*)	0.31 (*0.31*)<br>0.31 (*0.31*)
Macrolides/ lincosamides	Obs.:<br>Std.:	4.82 (*3.4*)<br>4.76 (*3.34*)	4.93 (*3.34*)<br>4.93 (*3.34*)	4.64 (*3.26*)<br>4.66 (*3.27*)	4.27 (*2.97*)<br>4.26 (*2.97*)	4.63 (*3.24*)<br>4.65 (*3.25*)	4.83 (*3.32*)<br>4.87 (*3.34*)
Tetracyclines	Obs.:<br>Std.:	0.69 (*0.37*)<br>0.68 (*0.36*)	1.19 (*0.65*)<br>1.18 (*0.64*)	1.16 (*0.65*)<br>1.1 (*0.62*)	0.73 (*0.41*)<br>0.74 (*0.42*)	0.73 (*0.4*)<br>0.73 (*0.4*)	0.8 (*0.43*)<br>0.83 (*0.44*)
Antifolates/ sulfonamides	Obs.:<br>Std.:	1.3 (*1.3*)<br>1.27 (*1.27*)	1.12 (*1.12*)<br>1.11 (*1.11*)	1.26 (*1.26*)<br>1.17 (*1.17*)	1.31 (*1.31*)<br>1.35 (*1.35*)	1.44 (*1.44*)<br>1.44 (*1.44*)	1.27 (*1.27*)<br>1.34 (*1.34*)

		Consumption in DDD/100PD (*RDD/100PD*)					
		Northwest	Northeast	Centre/East	Southeast	Southwest	West
Linezolid	Obs.:	0.62 (*0.62*)	0.64 (*0.64*)	0.68 (*0.68*)	0.77 (*0.77*)	0.68 (*0.68*)	0.62 (*0.62*)
	Std.:	0.63 (*0.63*)	0.63 (*0.63*)	0.65 (*0.65*)	0.77 (*0.77*)	0.68 (*0.68*)	0.63 (*0.63*)
Metroni-dazole	Obs.:	2.38 (*2.51*)	1.8 (*1.88*)	1.91 (*2.01*)	1.96 (*2.05*)	2.14 (*2.23*)	1.62 (*1.72*)
	Std.:	2.34 (*2.47*)	1.8 (*1.88*)	1.89 (*2*)	1.88 (*1.97*)	2.14 (*2.23*)	1.57 (*1.67*)

***Table S6**: Overview of observed (Obs.) and standardized (Std.) antibiotic use densities in DDD/100PD and RDD/100PD by region. Psychiatric/psychosomatic and paediatric wards are excluded from the calculations. Standardization of antibiotic use densities is performed based on the distribution of hospital size/bed count category in the overall study population of hospitals.*

Regression analyses

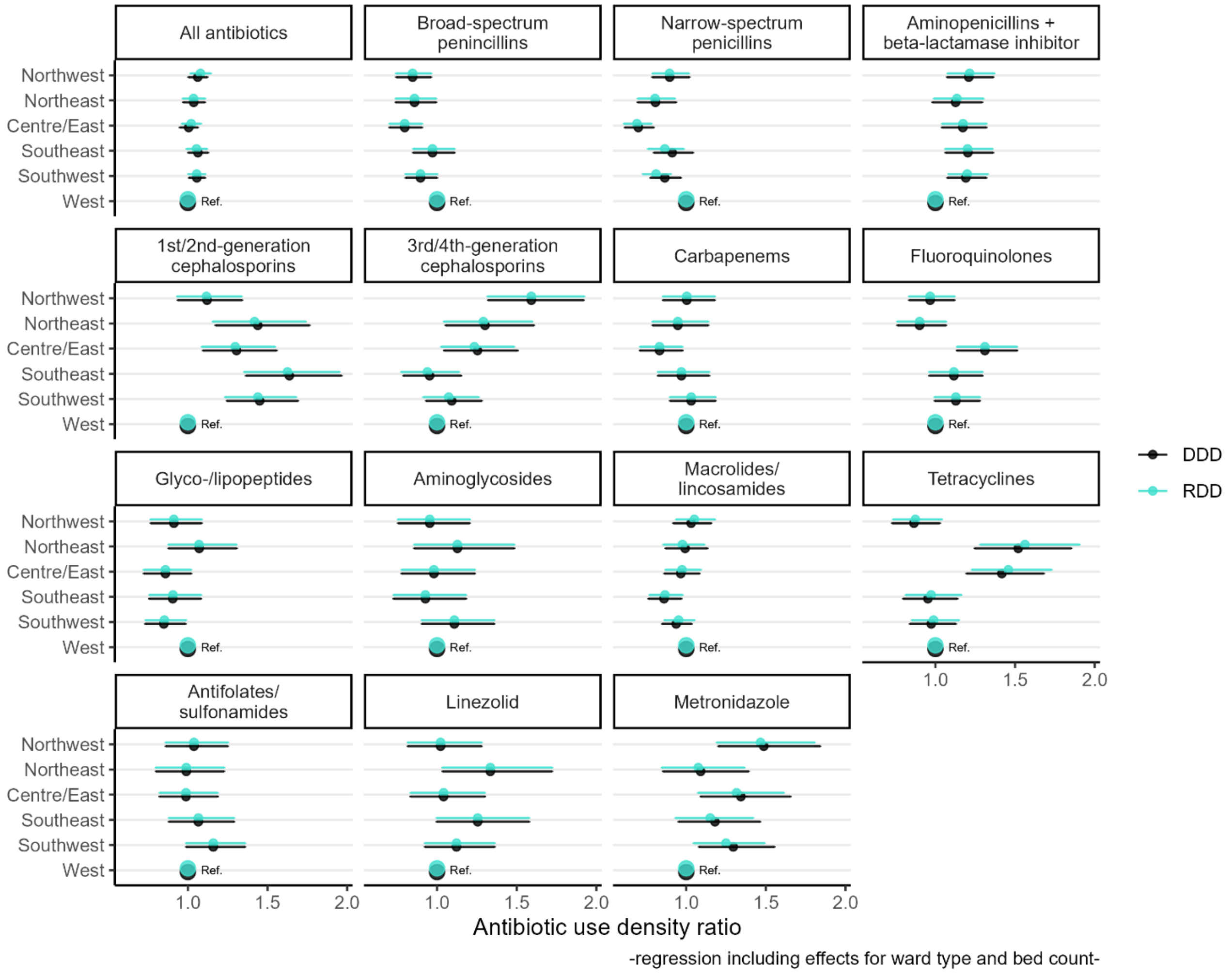

Figure S6: *Comparison of regional effects in the regression model depending on the outcome measure (DDD or RDD). Regression coefficients per region are shown for the zero-inflated Gamma mixed-effects regressions incorporating the covariates region, bed count, and ward type. Region West is chosen as the reference category as it has the largest number of hospitals in the sample. The regression models are based on data from 525 German non-university acute care hospitals.*

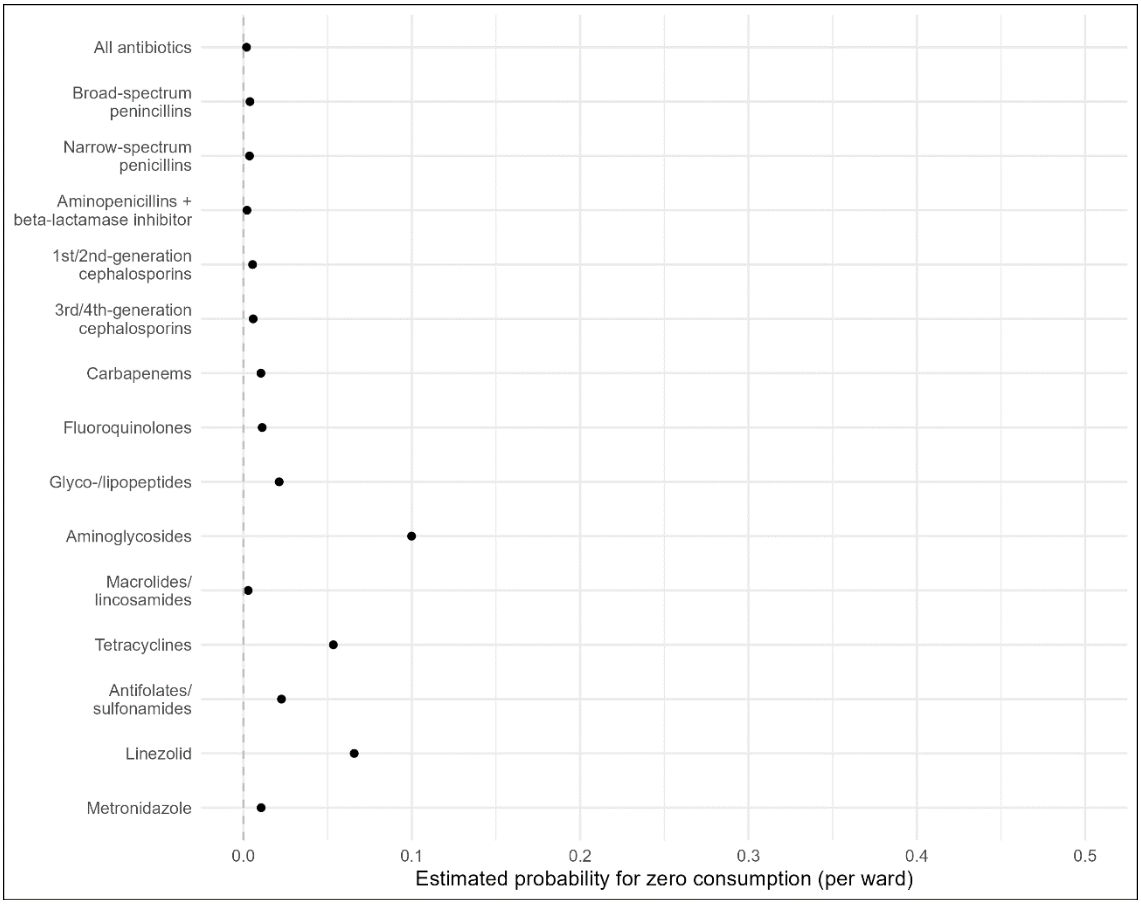

Figure S7: *Estimated probability for zero consumption based on zero-inflated Gamma regression models for the considered antibiotic groups.*

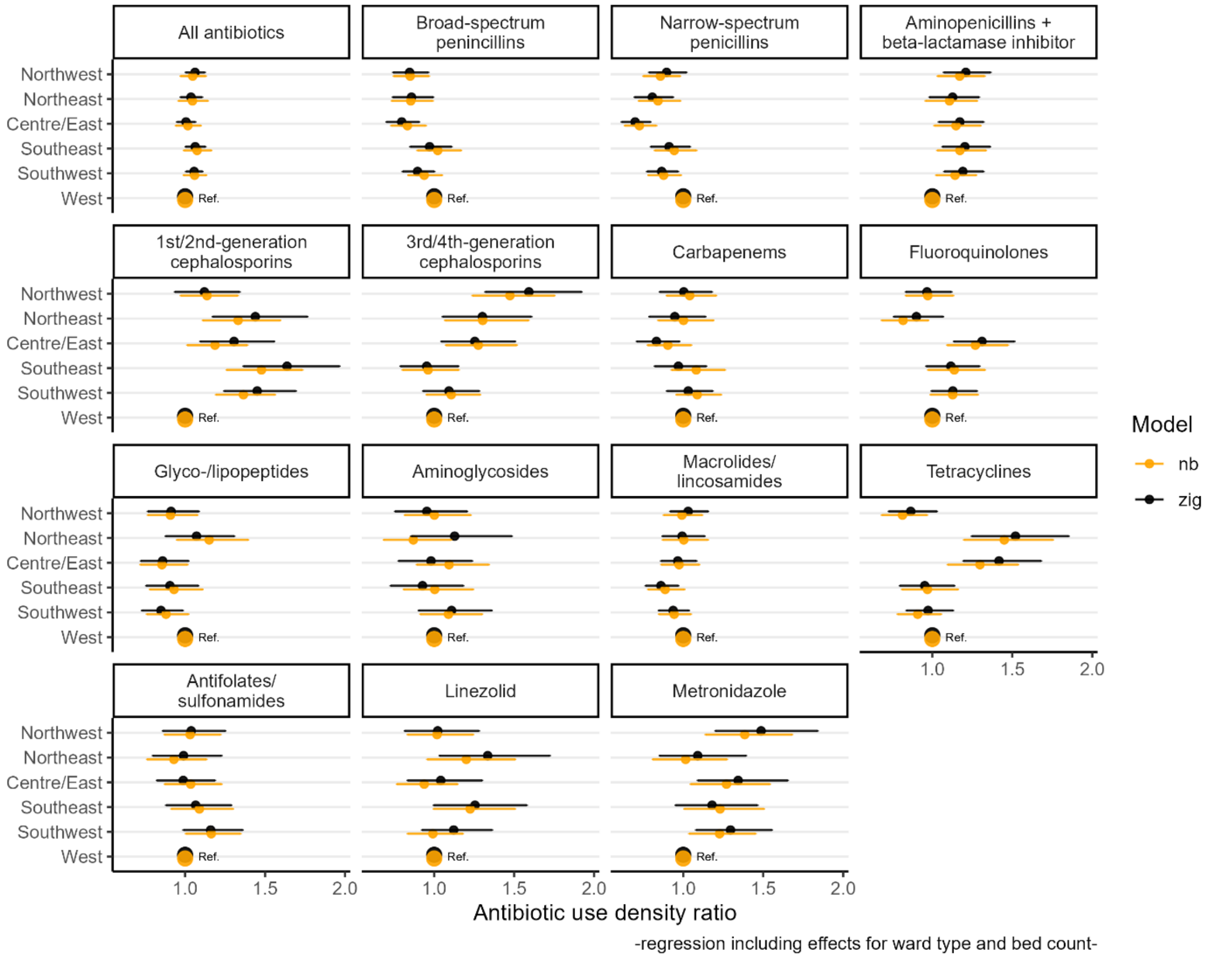

Figure S8: Regression coefficients for regional differences based on negative-binomial regression (nb) compared with coefficients based on zero-inflated Gamma regression (zig).

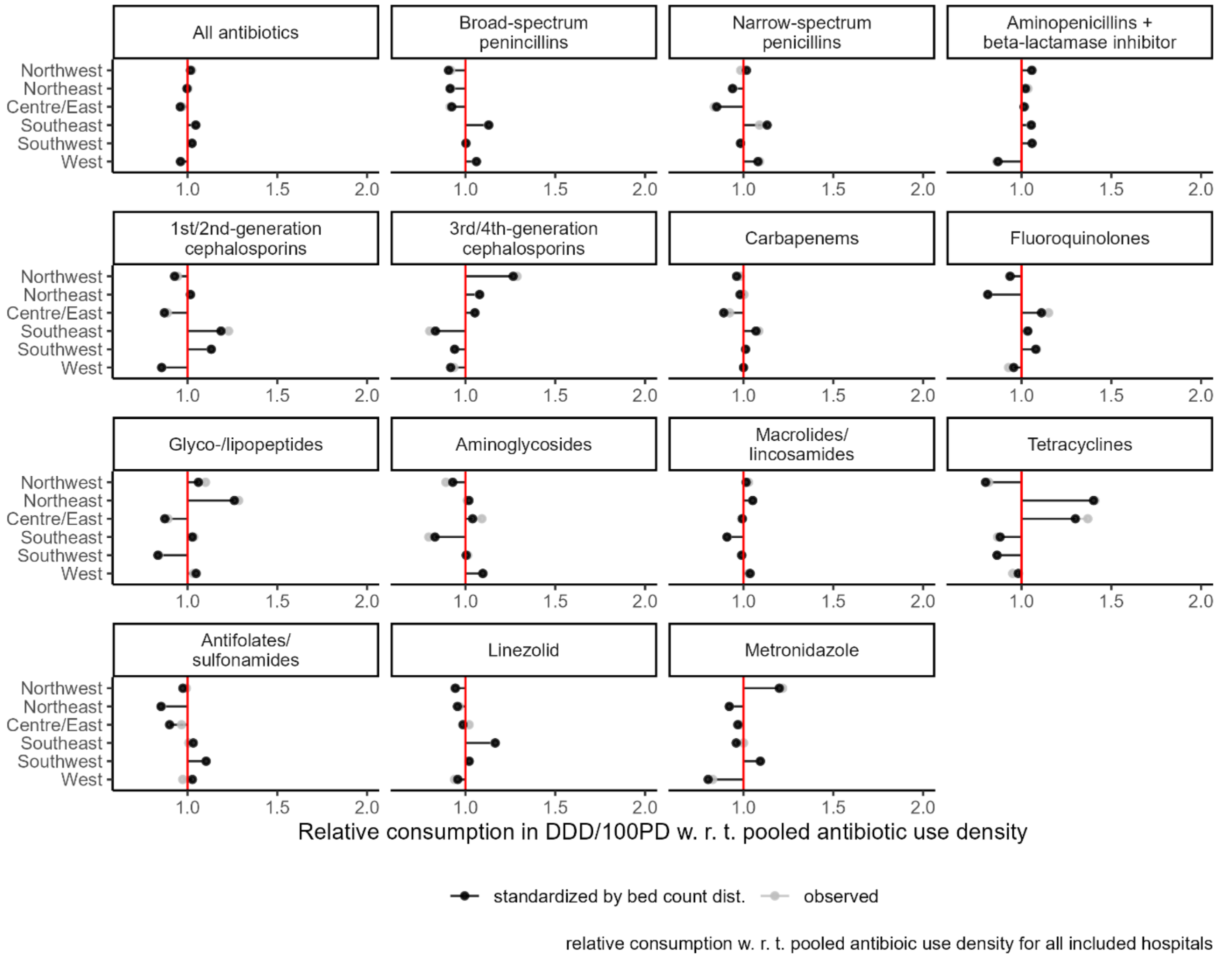

***Figure S9*: Relative deviations of observed (grey) and standardized (black) antibiotic use densities (DDD/100 PD) from the pooled antibiotic use density for 525 non-university acute care hospitals. The standardized densities are calculated by reweighting the observed antibiotic use density for each region to reflect the distribution of hospital sizes in the study population. Consumption densities are normalized such that 1 (red line) represents the pooled consumption density for all non-university acute care hospitals considered.*

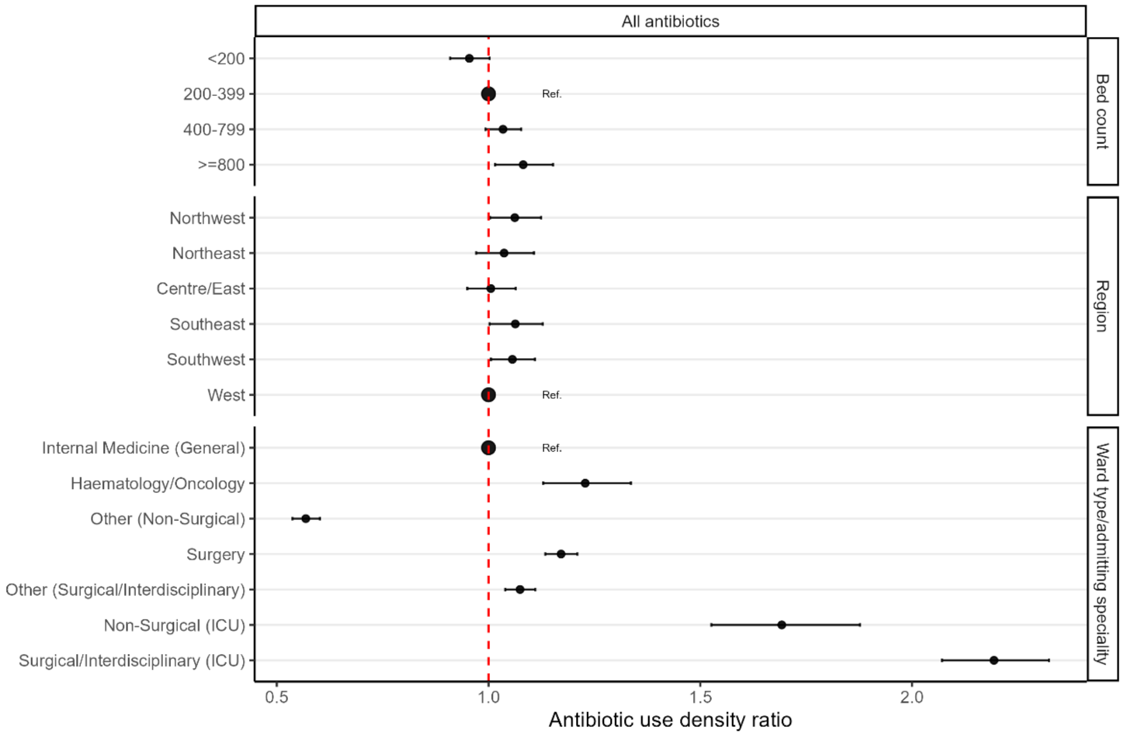

***Figure S10**: Full set of regression coefficients for zero-inflated Gamma regression for group of all antibiotics.*

	Estimated coefficients (with 95% confidence interval)
	All antibiotics
Region	
Northwest	1.06 (1-1.12)
Northeast	1.04 (0.97-1.11)
Centre/East	1.01 (0.95-1.06)
Southeast	1.06 (1-1.13)
Southwest	1.06 (1.01-1.11)
West	Reference
Bed count	
<200	0.95 (0.91-1)
200-399	Reference
400-799	1.03 (0.99-1.08)
≥800	1.08 (1.02-1.15)
Ward type/admitting specialty	
Internal Medicine (General)	Reference
Haematology/Oncology	1.23 (1.13-1.34)
Other (Non-Surgical)	0.57 (0.54-0.6)
Surgery	1.17 (1.13-1.21)
Other (Surgical/Interdisciplinary)	1.07 (1.04-1.11)
Non-Surgical (ICU)	1.69 (1.53-1.88)
Surgical/Interdisciplinary (ICU)	2.19 (2.07-2.32)

Table S7: *Full set of regression coefficients for zero-inflated Gamma regression for group of all antibiotics.*

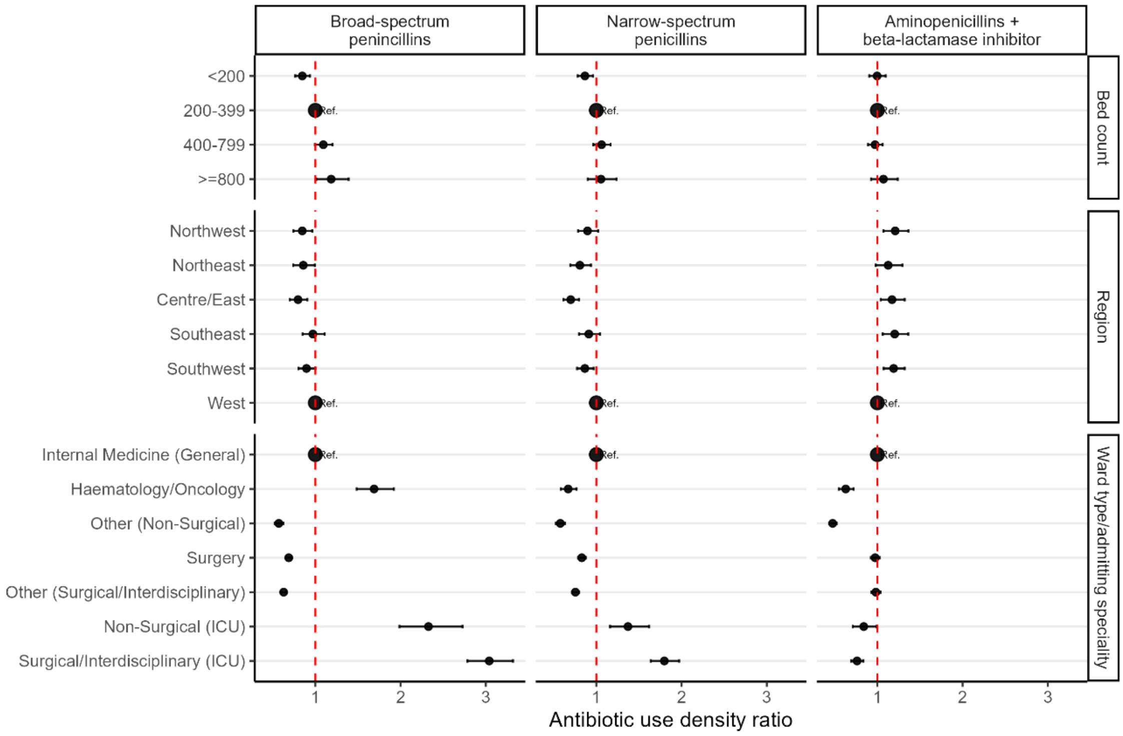

***Figure S11**: Full set of regression coefficients for zero-inflated Gamma regression for broad-spectrum penicillins, aminopenicillins with β -lactamase inhibitors (BLI), and narrow-spectrum penicillins.*

	Estimated coefficients (with 95% confidence interval)		
	Aminopenicilins /ß-lactamase inhibitor	Broad-spectrum penicillins	Narrow- spectrum penicillins
Region			
Northwest	1.21 (1.07-1.36)	0.85 (0.74-0.96)	0.9 (0.79-1.02)
Northeast	1.13 (0.98-1.29)	0.86 (0.74-0.99)	0.81 (0.69-0.94)
Centre/East	1.17 (1.04-1.32)	0.8 (0.7-0.91)	0.7 (0.61-0.8)
Southeast	1.2 (1.06-1.36)	0.97 (0.85-1.11)	0.91 (0.8-1.04)
Southwest	1.19 (1.07-1.32)	0.9 (0.8-1)	0.87 (0.77-0.97)
West	Reference	Reference	Reference
Bed count			
<200	1 (0.9-1.1)	0.85 (0.76-0.94)	0.87 (0.78-0.96)
200-399	Reference	Reference	Reference
400-799	0.97 (0.89-1.06)	1.09 (0.99-1.2)	1.06 (0.97-1.17)
≥800	1.07 (0.93-1.24)	1.19 (1.01-1.39)	1.05 (0.9-1.24)
Ward type/admitting specialty			
Internal Medicine (General)	Reference	Reference	Reference
Haematology/Oncology	0.63 (0.55-0.72)	1.69 (1.48-1.92)	0.67 (0.58-0.77)
Other (Non-Surgical)	0.48 (0.43-0.52)	0.57 (0.52-0.62)	0.58 (0.53-0.63)
Surgery	0.97 (0.92-1.03)	0.69 (0.65-0.72)	0.83 (0.79-0.87)
Other (Surgical/Interdisciplinary)	0.98 (0.93-1.04)	0.63 (0.6-0.66)	0.75 (0.71-0.8)
Non-Surgical (ICU)	0.84 (0.71-0.99)	2.33 (1.99-2.73)	1.37 (1.16-1.62)
Surgical/Interdisciplinary (ICU)	0.76 (0.7-0.84)	3.04 (2.78-3.32)	1.8 (1.64-1.97)

Table S8*: Full set of regression coefficients for zero-inflated Gamma regression for broad-spectrum penicillins, aminopenicillins with β -lactamase inhibitors, and narrow-spectrum penicillins.*

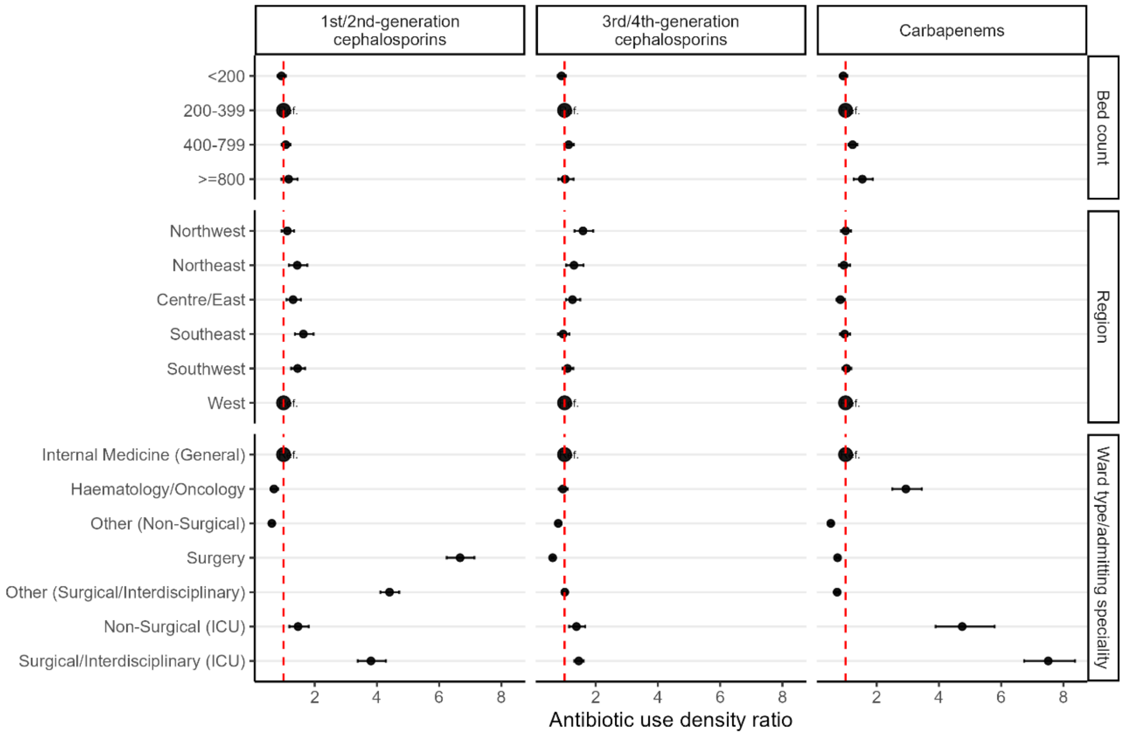

Figure S12: *Full set of regression coefficients for zero-inflated Gamma regression for cephalosporins and carbapenems.*

	Estimated coefficients (with 95% confidence interval)		
	1./2. generation cephalosporins	3./4. generation cephalosporins	Carbapenems
Region			
Northwest	1.12 (0.94-1.34)	1.59 (1.32-1.92)	1 (0.85-1.18)
Northeast	1.44 (1.17-1.76)	1.3 (1.05-1.61)	0.95 (0.79-1.14)
Centre/East	1.31 (1.09-1.56)	1.25 (1.04-1.51)	0.83 (0.71-0.98)
Southeast	1.64 (1.36-1.97)	0.95 (0.79-1.15)	0.97 (0.82-1.14)
Southwest	1.45 (1.24-1.69)	1.09 (0.93-1.28)	1.03 (0.9-1.18)
West	Reference	Reference	Reference
Bed count			
<200	0.93 (0.81-1.07)	0.9 (0.78-1.04)	0.93 (0.82-1.05)
200-399	Reference	Reference	Reference
400-799	1.07 (0.94-1.22)	1.13 (0.99-1.3)	1.23 (1.09-1.38)
≥800	1.16 (0.93-1.45)	1.02 (0.8-1.29)	1.54 (1.26-1.87)
Ward type/admitting specialty			
Internal Medicine (General)	Reference	Reference	Reference
Haematology/Oncology	0.69 (0.58-0.82)	0.95 (0.81-1.1)	2.94 (2.5-3.45)
Other (Non-Surgical)	0.62 (0.55-0.7)	0.8 (0.72-0.88)	0.52 (0.47-0.59)
Surgery	6.67 (6.24-7.13)	0.62 (0.58-0.66)	0.74 (0.7-0.79)
Other (Surgical/Interdisciplinary)	4.4 (4.11-4.72)	1.01 (0.95-1.07)	0.73 (0.68-0.78)
Non-Surgical (ICU)	1.47 (1.19-1.81)	1.38 (1.15-1.67)	4.75 (3.89-5.79)
Surgical/Interdisciplinary (ICU)	3.81 (3.38-4.29)	1.46 (1.31-1.61)	7.51 (6.74-8.37)

Table S9: *Full set of regression coefficients for zero-inflated Gamma regression for cephalosporins and carbapenems.*

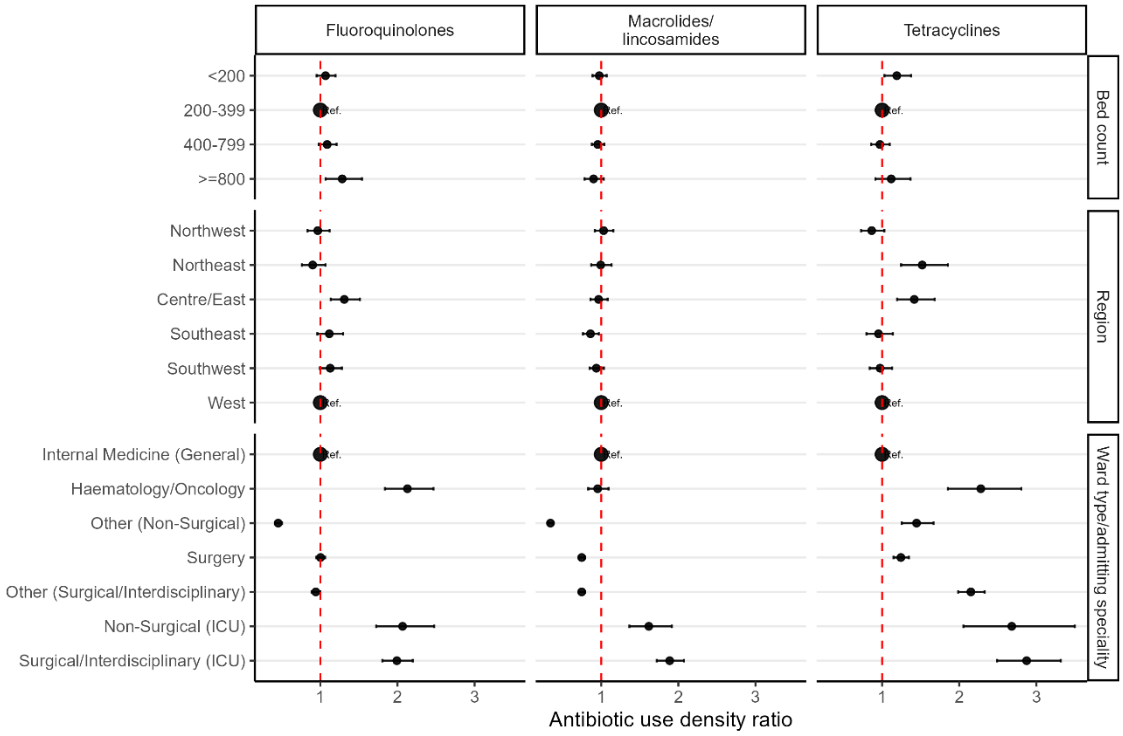

***Figure S13**: Full set of regression coefficients for zero-inflated Gamma regression for fluoroquinolones, macrolides, and lincosamides and tetracyclines.*

	Estimated coefficients (with 95% confidence interval)		
	Fluoroquinolones	Macrolides/lincosamides	Tetracyclines
Region			
Northwest	0.97 (0.83-1.12)	1.03 (0.92-1.16)	0.86 (0.73-1.03)
Northeast	0.9 (0.76-1.07)	0.99 (0.87-1.13)	1.52 (1.25-1.85)
Centre/East	1.31 (1.13-1.51)	0.97 (0.86-1.08)	1.42 (1.19-1.68)
Southeast	1.12 (0.96-1.3)	0.86 (0.76-0.97)	0.95 (0.8-1.14)
Southwest	1.13 (0.99-1.28)	0.94 (0.85-1.04)	0.97 (0.84-1.13)
West	Reference	Reference	Reference
Bed count			
<200	1.07 (0.95-1.2)	0.97 (0.89-1.07)	1.19 (1.03-1.38)
200-399	Reference	Reference	Reference
400-799	1.09 (0.98-1.21)	0.95 (0.88-1.04)	0.97 (0.86-1.1)
≥800	1.28 (1.07-1.54)	0.9 (0.78-1.03)	1.12 (0.91-1.37)
Ward type/admitting specialty			
Internal Medicine (General)	Reference	Reference	Reference
Haematology/Oncology	2.13 (1.84-2.47)	0.95 (0.83-1.09)	2.28 (1.85-2.81)
Other (Non-Surgical)	0.45 (0.41-0.5)	0.34 (0.31-0.38)	1.45 (1.26-1.67)
Surgery	1 (0.95-1.06)	0.75 (0.71-0.79)	1.24 (1.15-1.35)
Other (Surgical/Interdisciplinary)	0.94 (0.89-1)	0.75 (0.71-0.79)	2.15 (1.99-2.33)
Non-Surgical (ICU)	2.07 (1.72-2.48)	1.62 (1.36-1.92)	2.68 (2.05-3.5)
Surgical/Interdisciplinary (ICU)	1.99 (1.8-2.2)	1.89 (1.72-2.07)	2.87 (2.49-3.32)

***Table S10**: Full set of regression coefficients for zero-inflated Gamma regression for fluoroquinolones, macrolides, and lincosamides and tetracyclines.*

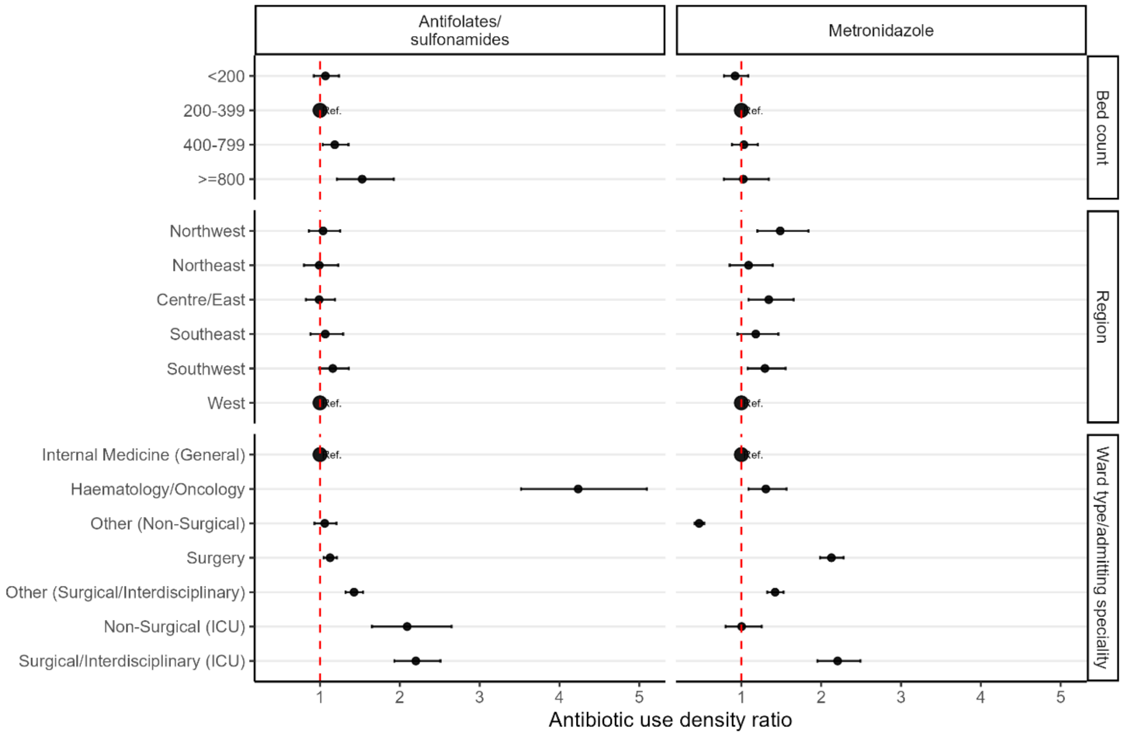

Figure S14: *Full set of regression coefficients for zero-inflated Gamma regression for antifolates/sulfonamides and metronidazole.*

	Estimated coefficients (with 95% confidence interval)	
	Antifolates /sulfonamides	Metronidazole
Region		
Northwest	1.04 (0.86-1.25)	1.49 (1.2-1.84)
Northeast	0.99 (0.8-1.23)	1.09 (0.85-1.39)
Centre/East	0.99 (0.82-1.19)	1.34 (1.09-1.65)
Southeast	1.07 (0.88-1.29)	1.18 (0.95-1.46)
Southwest	1.16 (0.99-1.36)	1.3 (1.08-1.55)
West	Reference	Reference
Bed count		
<200	1.07 (0.92-1.24)	0.92 (0.78-1.09)
200-399	Reference	Reference
400-799	1.18 (1.03-1.36)	1.03 (0.88-1.21)
≥800	1.53 (1.21-1.92)	1.02 (0.78-1.34)
Ward type/admitting specialty		
Internal Medicine (General)	Reference	Reference
Haematology/Oncology	4.23 (3.52-5.09)	1.31 (1.09-1.57)
Other (Non-Surgical)	1.06 (0.93-1.21)	0.47 (0.41-0.53)
Surgery	1.13 (1.05-1.21)	2.13 (1.99-2.28)
Other (Surgical/Interdisciplinary)	1.43 (1.32-1.54)	1.42 (1.32-1.53)
Non-Surgical (ICU)	2.09 (1.65-2.65)	1 (0.8-1.26)
Surgical/Interdisciplinary (ICU)	2.2 (1.93-2.51)	2.21 (1.95-2.49)

***Table S11**: Full set of regression coefficients for zero-inflated Gamma regression for antifolates/sulfonamides and metronidazole.*

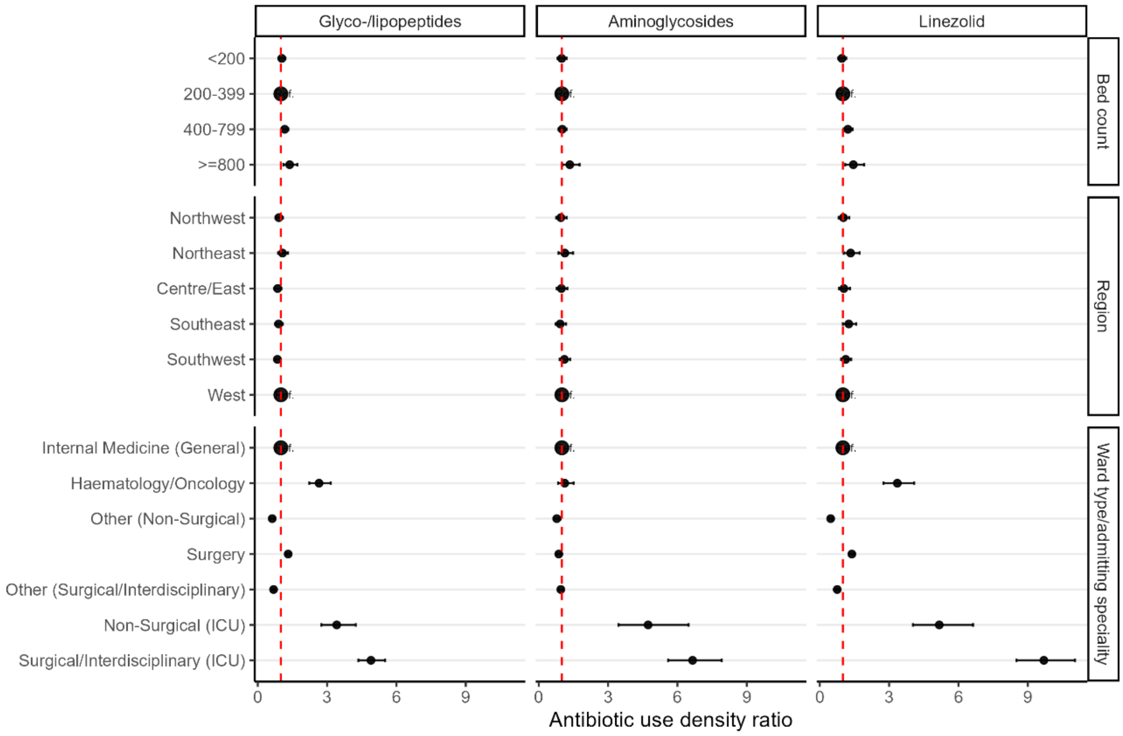

***Figure S15**: Full set of regression coefficients for zero-inflated Gamma regression for glycopeptides, aminoglycosides, and linezolid.*

	Estimated coefficients (with 95% confidence interval)		
	Aminoglycosides	Glyco-/lipopeptides	Linezolid
Region			
Northwest	0.95 (0.75-1.21)	0.91 (0.77-1.09)	1.02 (0.82-1.28)
Northeast	1.13 (0.86-1.49)	1.07 (0.88-1.31)	1.33 (1.03-1.72)
Centre/East	0.98 (0.78-1.24)	0.86 (0.72-1.02)	1.04 (0.83-1.3)
Southeast	0.93 (0.73-1.18)	0.91 (0.76-1.08)	1.26 (1-1.58)
Southwest	1.11 (0.9-1.36)	0.85 (0.73-0.99)	1.12 (0.92-1.36)
West	Reference	Reference	Reference
Bed count			
<200	0.99 (0.81-1.21)	1.04 (0.91-1.19)	0.96 (0.8-1.14)
200-399	Reference	Reference	Reference
400-799	1.01 (0.86-1.2)	1.17 (1.03-1.33)	1.22 (1.03-1.43)
≥800	1.34 (1.02-1.77)	1.38 (1.11-1.71)	1.45 (1.1-1.92)
Ward type/admitting specialty			
Internal Medicine (General)	Reference	Reference	Reference
Haematology/Oncology	1.12 (0.84-1.51)	2.65 (2.23-3.16)	3.35 (2.76-4.08)
Other (Non-Surgical)	0.78 (0.64-0.94)	0.63 (0.55-0.71)	0.47 (0.4-0.55)
Surgery	0.87 (0.78-0.96)	1.31 (1.23-1.41)	1.39 (1.28-1.5)
Other (Surgical/Interdisciplinary)	0.95 (0.86-1.06)	0.68 (0.64-0.74)	0.75 (0.69-0.82)
Non-Surgical (ICU)	4.73 (3.45-6.48)	3.42 (2.75-4.24)	5.17 (4.03-6.63)
Surgical/Interdisciplinary (ICU)	6.65 (5.6-7.91)	4.9 (4.35-5.51)	9.69 (8.51-11.04)

***Table S12**: Full set of regression coefficients for zero-inflated Gamma regression for glycopeptides, aminoglycosides, and linezolid.*
